# Advances of Functional Two-Dimensional Nanomaterials in the Treatment of Oral Diseases

**DOI:** 10.3390/bioengineering12101021

**Published:** 2025-09-25

**Authors:** Ziyi Xu, Rong Meng, Yue Wang, Yuxuan Sun, Jiao Qiao, Yang Yao, Qiang Peng

**Affiliations:** State Key Laboratory of Oral Diseases, National Center for Stomatology, National Clinical Research Center for Oral Diseases, West China Hospital of Stomatology, Sichuan University, Chengdu 610041, China

**Keywords:** 2D nanomaterials, oral diseases, antimicrobial, tissue engineering, immunomodulation

## Abstract

Two-dimensional (2D) nanomaterials have attracted growing attention in the field of oral medicine due to their unique physicochemical properties, including high surface area, adjustable surface chemistry, and exceptional biocompatibility. In recent years, a variety of 2D materials, including graphene-based nanomaterials, black phosphorus nanosheets, MXenes, layered double hydroxides (LDHs), transition metal dichalcogenides (TMDs), 2D metal–organic frameworks (MOFs), and polymer-based nanosheets, have been extensively explored for the treatment of oral diseases. These functional materials demonstrate multiple therapeutic capabilities, such as antibacterial activity, reactive oxygen species (ROS) scavenging, anti-inflammatory modulation, and promotion of tissue regeneration. In this review, we systematically summarize the recent advances of 2D nanomaterials in the treatment of common oral diseases such as dental caries, periodontitis, oral cancer and peri-implantitis. The underlying therapeutic mechanisms are also summarized. Challenges for clinical translation of these nanomaterials and the possible solutions are discussed as well.

## 1. Introduction

Oral diseases are among the most prevalent chronic diseases worldwide, encompassing a wide range of diseases such as dental caries, periodontitis, oral squamous cell carcinoma, and peri-implantitis. These diseases significantly impair essential functions including mastication, speech, and facial aesthetics, while also exerting considerable impact on systemic health [1]. According to the data released by World Health Organization, approximately 3.5 billion people suffer from oral diseases, posing a significant burden on global health [2]. Although conventional therapies—such as mechanical debridement, antibiotic therapy, surgical interventions, and restorative reconstruction—are widely applied in clinical settings, critical limitations remain, such as disease recurrence, antibiotic resistance, and insufficient regenerative capacity [3]. For instance, periodontitis, a chronic condition driven by both immune and infectious mechanisms, cannot be effectively managed through antimicrobial treatment alone, as immune modulation and tissue regeneration are also required [4]. Similarly, high recurrence rates and collateral damage to healthy tissues in oral cancer treatment call for more precise and minimally invasive therapeutic strategies [5].

In recent years, the development of nanotechnology and biomaterials has opened up new possibilities for precision intervention in oral diseases [6,7]. Among these innovations, two-dimensional (2D) nanomaterials have attracted considerable attention due to their unique physicochemical properties [8]. Compared to their zero-dimensional (like quantum dots) or bulk three-dimensional counterparts (like bulk crystals), 2D materials exhibit ultrathin layered architectures, exceptionally high surface-area-to-volume ratios, outstanding surface modifiability, and strong responsiveness to optical, thermal, and electromagnetic stimuli [9,10]. These features endow them with superior drug-loading efficiency, enhanced adaptability at biological interfaces, and efficient photothermal/photodynamic conversion [11]. Furthermore, their intimate interactions at the nanoscale with cellular membranes, bacterial biofilms, and extracellular matrix components enable 2D nanomaterials to exert multifunctional and synergistic effects—such as antibacterial, anti-inflammatory, ROS-scavenging, immunomodulatory, and tissue-regenerative actions—making them especially suitable for multitargeted therapies in complex microenvironments [12,13].

To date, a broad range of 2D nanomaterials—including graphene-based materials, black phosphorus nanosheets (BP), MXenes, layered double hydroxides (LDHs), transition metal dichalcogenides (TMDs), and two-dimensional metal–organic frameworks (2D MOFs)—have been extensively studied in both fundamental and applied research across multiple oral diseases [14,15,16]. These materials have been harnessed to construct photo-responsive therapeutic platforms for noninvasive, localized bacterial elimination or tumor ablation. They also serve as drug and gene delivery carriers, enabling site-specific and controlled release. In addition, 2D nanomaterials have been used to modify implant surfaces, enhance osteoblast and fibroblast adhesion, and facilitate osseointegration and soft tissue sealing [17]. Moreover, several 2D materials have demonstrated immunomodulatory capabilities, including the regulation of macrophage polarization, suppression of pro-inflammatory cytokines, and promotion of angiogenesis—offering novel avenues for inflammation resolution and tissue regeneration in oral lesions [18].

**Figure 1:**
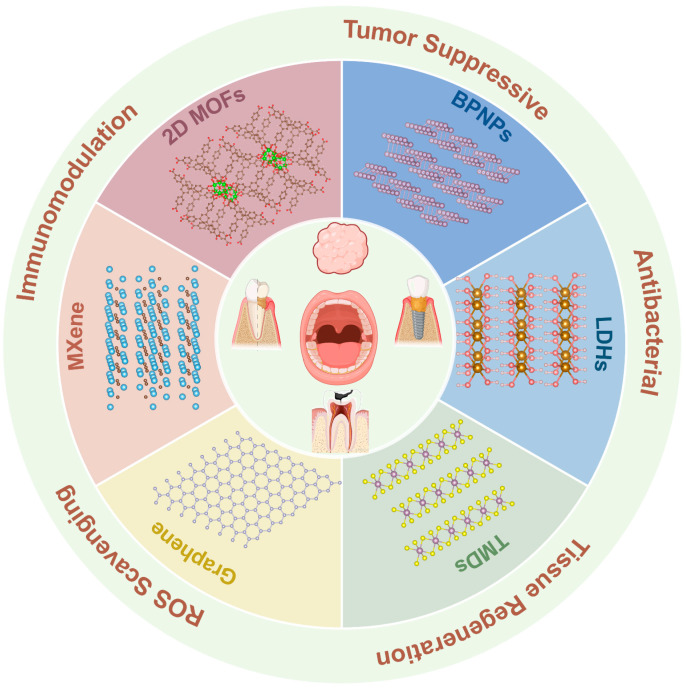
Common types of two-dimensional nanomaterials and their therapeutic applications in oral diseases.

This review aims to systematically summarize the recent advances in the application of 2D nanomaterials for the treatment of oral diseases (Figure 1). We classify current research strategies based on material types and elaborate on their therapeutic mechanisms and functions, including antibacterial and anti-inflammatory activities, photothermal/photodynamic therapies, ROS scavenging, drug delivery, and tissue repair. Furthermore, we critically discuss the major challenges hindering their clinical translation and envision future directions for the development of multifunctional, controllable, and clinically translatable therapeutic platforms in precision oral medicine.

## 2. Unique Advantages Beneficial for Oral Treatment

### 2.1. High Specific Surface Area

The monolayer or few-layer structure of two-dimensional (2D) materials endows them with an exceptionally high specific surface area, enabling a greater number of accessible surface sites per unit mass. This structural advantage significantly enhances their performance in drug loading, surface modification, and interactions with cellular or bacterial targets [19]. In the context of oral therapy, the large surface area of 2D materials facilitates stronger adhesion to hard dental tissues, periodontal soft tissues, or implant surfaces, thereby improving therapeutic efficacy and prolonging local retention [20]. Moreover, the increased surface area promotes intimate contact with both bacteria and host cells, contributing to enhanced antibacterial activity and therapeutic outcomes.

Structurally, 2D materials feature atomic-level thickness and extended lateral dimensions, forming an “ultrathin planar” geometry that exposes most atoms at the surface. This overcomes the limitation of bulk materials whose interior atoms cannot participate in interfacial reactions and results in higher activity per unit mass [21].

For drug delivery, Graphene Oxide (GO), Black Phosphorus (BP), and Transition Metal Dichalcogenides (TMDs) efficiently load antibacterial, anti-inflammatory, and photosensitizing agents via π–π stacking/van der Waals forces and covalent/coordination interactions [22]. In addition, their layered structure and high surface area also confer excellent “membrane-wrapping/adhesion” capability, aiding disruption of mature biofilms and suiting deep periodontal pockets or subgingival lesions [23].

### 2.2. Surface Modifiability and Functionalization

Two-dimensional (2D) nanomaterials inherently possess abundant reactive groups on their surfaces and edges, such as hydroxyl, carboxyl, phosphate, and thiol groups, and can be further chemically functionalized to introduce specific active sites that facilitate drug delivery, targeted recognition, inflammation modulation, and tissue regeneration. Functional molecules can be coupled by covalent linkages (amide/ester/thioether) or non-covalent interactions (electrostatic, π–π stacking, hydrogen bonding, hydrophobic) [24]. For example, oxygenated groups in GO enable covalent conjugation with amine-containing drugs, peptides, or macromolecules, while the aromatic domains of GO permit stable π–π adsorption of small molecules and photosensitizers [25]. Surfaces can also be engineered to respond to oral pathological cues (e.g., accelerated release in acidic environments) and to incorporate targeting ligands such as RGD peptides or sugars for selective binding to gingival tissues, osteogenic cells, or pathogens, thereby improving therapeutic precision [26]. These surface engineering strategies allow 2D materials to serve as multifunctional therapeutic platforms with antibacterial, anti-inflammatory, osteoinductive, and mucosal regenerative capabilities [27].

### 2.3. Photothermal and Photodynamic Activity

Owing to unique band structures, high surface area, and abundant active sites, 2D materials efficiently absorb and convert near-infrared (NIR) light, making them suitable for non-invasive, localized PTT/PDT in oral disease treatment. In PTT, metallic 2D materials rely on localized surface plasmon resonance (LSPR) to dissipate light energy as heat [28,29]. Semiconductor materials like MoS_2_ and BP generate heat via non-radiative relaxation of photoexcited carriers [30]. In PDT, tunable/narrow bandgaps facilitate efficient excitation and carrier separation, with energy transfer to O_2_ generating ROS such as ^1^O_2_, O_2_^−^, and ·OH [31]. In addition, synergistic PTT/PDT can enhance local perfusion and ROS diffusion via heating, while increased membrane permeability expands PDT’s effective range; light-triggered release and coupled thermal/oxidative stress have shown precise therapeutic potential in oral squamous cell carcinoma and biofilm-associated infections [32].

### 2.4. High Mechanical Stability

Two-dimensional (2D) nanomaterials exhibit outstanding mechanical stability suited to the oral environment, which features frequent masticatory loading, continuous saliva flow, and pH fluctuations. Representative 2D systems, such as graphene, MXenes, and BN, possess dense lattices and robust interlayer interactions, yielding high tensile strength, elastic modulus, and fracture resistance for oral coatings, membranes, and scaffolds [33].

For instance, monolayer graphene boasts a tensile strength of up to 130 GPa and a Young’s modulus of approximately 1 TPa, making it one of the strongest materials known to date [34]. When incorporated into nanocomposites as a reinforcing phase, graphene and its oxidized derivatives (GO) can significantly enhance tear and fracture resistance. In applications such as periodontal regeneration scaffolds, hydrogel carriers, and oral adhesive membranes, these materials help maintain structural integrity under mechanical stress, thereby ensuring sustained biological function [35]. MXenes combine strength with flexibility; their layered architecture buffers microscale stress and surface –OH/–F groups enable robust polymer/protein networks, supporting long-term mechanical and functional stability in pocket-sealing membranes, root coverings, and antibacterial implant coatings [36].

### 2.5. Endogenous Bioactivity

#### 2.5.1. Antibacterial Effects and Mechanisms

With layered structures, high surface area, and rich functionality, 2D nanomaterials achieve broad-spectrum, efficient, and low-resistance antibacterial effects via endogenous and stimulus-responsive routes. Endogenously, ultrathin and sharp nanosheets physically disrupt bacterial membranes, causing leakage of proteins, DNA, and phospholipids [37]. Concurrently, ROS-dependent and ROS-independent oxidative damage targets membranes, proteins, and genetic material and compromises biofilm integrity [38]. Exogenously, PTT converts NIR light into heat for rapid bactericidal action. PDT generates ROS upon light excitation to oxidize and kill bacteria [5]. Other reports describe microwave dielectric heating or additional physical stimuli to treat deep infections or reduce drug burden [11].

#### 2.5.2. Immunomodulatory Effects and Mechanisms

Chronic oral inflammation is characterized by ROS accumulation, overexpression of pro-inflammatory cytokines, and aberrant immune polarization. 2D materials can rebalance redox state and modulate inflammatory signaling and immune-cell behavior without relying on conventional anti-inflammatories. For example, BP donates electrons to scavenge ROS, alleviating local oxidative stress [39]. Lower ROS levels help restrain NF-κB/MAPK cascades and pro-inflammatory cytokine release. At the cellular level, 2D materials can shift macrophages from M1 toward pro-regenerative phenotypes, and MXene systems can stabilize M2-like states via metabolic reprogramming, enabling sustained regulation of the inflammatory microenvironment [40].

#### 2.5.3. Osteogenic Promotion and Underlying Mechanisms

2D nanomaterials have shown great promise in alveolar bone defect repair, enhancing osseointegration and periodontal regeneration, with osteogenesis arising from a structure cues–signaling activation–microenvironment modulation triad. Surface roughness, hydrophilicity, and charge support MSC adhesion/spreading and cytoskeletal remodeling, activating mechanotransduction. At the molecular level, materials engage osteogenic pathways including Wnt/β-catenin, TGF-β/BMP, PI3K/Akt, and MAPK [41]. Unlike inert scaffolds, BP can release phosphate ions during degradation that participate in hydroxyapatite (HA) mineralization, conferring intrinsic osteoinductivity [42]. Multi-ion strategies (like synergy with Zn^2+^, Mg^2+^, or Ca^2+^/Si^4+^) can further enhance osteoinduction and regenerative repair [43].

#### 2.5.4. Promotion of Tissue Repair

Ultrathin, large-lateral 2D materials act as biomimetic scaffolds that enhance epithelial/fibroblast adhesion, migration, and proliferation, accelerating wound closure and mucosal regeneration. Beyond structural support, they modulate early healing by promoting M2 macrophage polarization and suppressing IL-6/TNF-α, while their high surface area stabilizes pro-healing factors to improve local bioavailability and angiogenesis [44].

Representative systems illustrate these effects: BP nanosheets activate JAK–STAT–OAS to boost endothelial metabolism and angiogenesis, expediting tissue regeneration [45], and Nb_2_C MXene scaffolds under mild photothermal cues co-activate HIF-1/STAT3/VEGF and PI3K/Akt, driving H-type capillary formation and vascularized bone regeneration [46].

## 3. Synthesis of Two-Dimensional Nanomaterials

**Figure 2:**
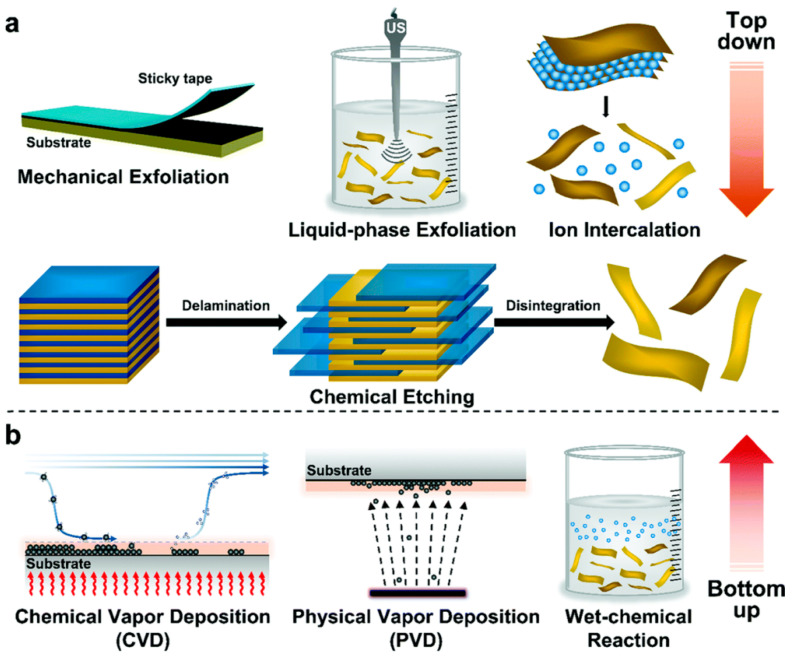
Schematic diagrams of the synthesis strategies of 2D biomaterials: (**a**) top-down fabrication and (**b**) bottom-up synthesis [47]. Copyright 2021, Royal Society of Chemistry (RSC).

As illustrated in Figure 2, the synthesis of 2D nanomaterials (2DNMs) is a crucial step in harnessing their unique structural and functional advantages, especially for biomedical applications in complex microenvironments such as the oral cavity. These synthesis strategies are primarily categorized into two approaches: “top-down” and “bottom-up” [47]. The former involves exfoliating layered bulk materials into two-dimensional structures through physical or chemical means, while the latter constructs 2D materials from atoms, ions, or molecules via chemical assembly. Each approach has its own merits and limitations in terms of structural integrity, yield, scalability, and functional integration, and the method of choice depends on the target material and its intended application.

### 3.1. Top-Down Methods

#### 3.1.1. Mechanical Exfoliation

Mechanical exfoliation is one of the earliest methods developed for obtaining two-dimensional nanomaterials. A representative example is the so-called “Scotch tape method,” in which bulk graphite is repeatedly peeled using adhesive tape to isolate single-layer or few-layer graphene [48]. This method is simple to operate, low-cost, and requires minimal reaction conditions, enabling the production of highly crystalline and nearly defect-free 2D materials under ambient temperature and pressure. It is commonly used to prepare ideal model materials for fundamental research. The basic principle involves applying external forces—such as shear, friction, or adhesion—to overcome the van der Waals interactions between layers in the bulk material, thereby achieving separation into ultrathin sheets. In addition to the traditional manual scotch tape method, a range of advanced mechanical exfoliation strategies have been developed in recent years, including in situ adhesive transfer under ultra-high vacuum, metal-assisted exfoliation, multistep peeling designs, and automated micromechanical systems, significantly improving the yield, layer size uniformity, and cleanliness of 2D material production [49].

Mechanical exfoliation is suitable for materials with naturally layered structures, such as graphite, black phosphorus, and TMDs, and can preserve the original crystal structure and electronic properties. However, its limitations—including low yield, poor size control, and difficulty in scale-up—render it unsuitable for large-scale production. Therefore, despite its continued importance in scientific research, mechanical exfoliation is primarily employed as a complementary or validation method rather than a practical manufacturing approach [50].

#### 3.1.2. Liquid-Phase Exfoliation

Liquid-phase exfoliation is an extensively applied top-down approach for preparing 2D nanosheets from layered materials. It offers several advantages, including ease of operation, compatibility with a wide range of material systems, and suitability for large-scale production. This method is particularly well-suited for the mass synthesis of various 2D materials such as graphene, BP, TMDs, and MXenes. The fundamental principle involves dispersing bulk layered materials into a selected solvent, followed by physical treatments such as ultrasonication or high-shear mixing to overcome interlayer van der Waals forces, thereby delaminating the layers into single or few-layer nanosheets [51]. To enhance exfoliation efficiency and maintain dispersion stability, it is essential to choose solvents with surface energies that match the material, or to introduce surfactants and polymer stabilizers to prevent nanosheet aggregation and restacking [52].

Liquid-phase exfoliation is characterized by its broad applicability and cost-effectiveness. It is particularly advantageous for producing large-quantity dispersions of 2D materials with sizable lateral dimensions and high yield, making it highly suitable for downstream processes such as spraying, self-assembly, and fabrication of composite membranes. The resulting products typically exhibit high specific surface areas and excellent processability, enabling their wide application in constructing platforms for oral disease treatment, such as antibacterial coatings, periodontal dressings, and drug delivery systems [53,54].

However, this method also presents certain limitations. The produced nanosheets often show broad distributions in thickness and lateral size, and ultrasonication may introduce structural defects. Moreover, exfoliation efficiency is influenced by multiple factors, including material type, solvent selection, and processing time, all of which require continuous optimization and validation [55].

#### 3.1.3. Chemical Intercalation and Exfoliation Method

The chemical intercalation and exfoliation method introduces intercalating agents into layered materials to disrupt their interlayer interactions, thereby enabling exfoliation of individual layers. This approach offers advantages in enhancing yield and structural tunability, and is particularly suitable for materials with strong interlayer bonding that are difficult to exfoliate physically. In practical operations, intercalating agents such as acids, bases, ionic liquids, small organic molecules, or metal ions are typically introduced into layered structures. These agents insert into the interlayer space, increase the interlayer distance, and weaken van der Waals forces, thereby facilitating the exfoliation of nanosheets through auxiliary methods such as ultrasonication, hydration, or heating [56]. The type and mechanism of the intercalating agent directly influence the exfoliation efficiency, nanosheet thickness, and the nature of surface functional groups. This method is often combined with reduction or surface modification steps to achieve multifunctional regulation of the material [57]. The resulting 2D nanosheets are rich in functional groups, facilitating subsequent drug loading, biological targeting, and both covalent and non-covalent modifications. This makes them particularly suitable for applications in the complex oral microenvironment, such as antibacterial therapy, tissue regeneration, and controlled drug delivery [58]. However, it should be noted that the intercalation and oxidation processes may introduce impurities or structural defects, thus necessitating post-treatment purification and stringent quality control [59].

### 3.2. Bottom-Up Methods

Unlike the Top-Down strategies that rely on exfoliating bulk materials, Bottom-Up methods start from atomic, ionic, or molecular precursors and gradually construct two-dimensional structures through controlled chemical or physical processes. These methods offer greater tunability at the atomic level, enabling precise control over the number of layers, defect density, morphology, doping, and surface chemistry. As such, they serve as powerful tools for the fabrication of structurally uniform and functionally designed two-dimensional nanomaterials (2DNMs), providing a foundational approach for targeted material design and multifunctional integration [60].

#### 3.2.1. Chemical Vapor Deposition (CVD)

Chemical Vapor Deposition is one of the most established and widely used Bottom-Up techniques, particularly for the synthesis of high-quality graphene, TMDs, and hexagonal boron nitride (h-BN). In this method, gaseous precursors containing the target elements (such as CH_4_, Mo(CO)_6_, or H_2_S) are introduced into a reaction chamber. Under high temperatures and inert or reducing gas atmospheres, these precursors decompose or react on the surface of catalytic or inert substrates, leading to the layer-by-layer growth of 2D films [61].

By tuning reaction parameters such as temperature, gas flow rates, precursor concentration, substrate type, and cooling rate, one can precisely control the thickness, crystal orientation, and growth rate of the resulting materials [62]. CVD-derived films are known for their low defect density and superior electrical and optical properties, making them ideal candidates for high-performance applications such as flexible electronics, field-effect transistors, and biosensors [63]. However, challenges remain in terms of high equipment cost, harsh synthesis conditions, limited precursor selection, and incompatibility with flexible or bioresorbable substrates, which hinder their broader application in clinical or biomedical fields [64].

#### 3.2.2. Physical Vapor Deposition (PVD)

Physical Vapor Deposition is another important Bottom-Up thin-film synthesis technique. It involves converting solid precursors into the vapor phase through heating, laser evaporation, or plasma bombardment, followed by condensation onto a substrate surface [65]. The entire process is purely physical and does not involve chemical reactions. Common PVD techniques include thermal evaporation, electron-beam evaporation, magnetron sputtering, and molecular beam epitaxy (MBE).

PVD is applicable to the preparation of various layered 2D materials such as graphene, multilayer hexagonal boron nitride (h-BN), MoS_2_, TiS_2_, and NbSe_2_ [66]. Its advantages include a simple and clean process that yields high-purity films and excellent control over film thickness and uniformity, making it suitable for large-area deposition and scalable manufacturing [67]. Notably, magnetron sputtering enables film growth at relatively low temperatures, avoiding thermal degradation of the material and making it ideal for fabricating flexible films with good biocompatibility [68].

Despite its advantages, PVD still faces certain limitations in the synthesis of 2D materials. Compared to chemical vapor deposition (CVD), it remains less effective in terms of selective monolayer growth, precise control over atomic-layer thickness, and crystal quality regulation [69]. Additionally, many PVD processes rely heavily on high-vacuum systems and high-purity precursors, and the growth rate is relatively slow. The range of materials that can be processed is also limited. Therefore, post-deposition treatments such as annealing, surface reconstruction, or the application of external energy fields are often necessary to improve film uniformity and crystallinity, enabling better structural and functional control of 2D materials [70].

#### 3.2.3. Hydrothermal/Solvothermal Synthesis

Hydrothermal and solvothermal methods are mild and efficient strategies for synthesizing 2D materials, typically carried out in sealed autoclaves using water or organic solvents under moderate temperature and pressure conditions (100–250 °C) [71]. These approaches are not only suitable for preparing a wide range of 2D materials, such as oxides, sulfides, phosphides, and certain two-dimensional metal–organic frameworks, but are also frequently employed to construct uniform and compact nanocoatings. Their advantages include low equipment cost, mild reaction conditions, and excellent scalability and tunability [72].

Reaction parameters such as time, temperature, pH, solvent polarity, and the type of surfactant or additive can be finely adjusted to control morphology, thickness, lateral dimensions, crystallinity, and surface functionalities [73]. Selective adsorption of additives (e.g., PVP, citric acid) on specific crystal facets can suppress vertical growth and promote lateral expansion, thereby favoring 2D structure formation [74]. The resulting materials typically exhibit good dispersibility and biocompatibility, making them suitable for applications in drug delivery, antibacterial coatings, and tissue engineering scaffolds. Nevertheless, the reaction mechanisms involved are often complex, and challenges remain in achieving high product purity and uniformity. Future advances will require the integration of in-situ characterization techniques and kinetic modeling to optimize synthesis pathways for reproducible and scalable production.

## 4. Application in Oral Diseases

### 4.1. Graphene and Its Derivatives

Graphene is a 2D nanomaterial composed of a single layer of sp^2^-hybridized carbon atoms arranged in a hexagonal honeycomb lattice. Since its first successful isolation through mechanical exfoliation by Geim and Novoselov in 2004, graphene has rapidly garnered widespread attention in material science, biomedicine, and energy fields due to its exceptional electrical conductivity, thermal conductivity, high specific surface area, and remarkable mechanical strength. To enhance the processability, dispersibility, and functional adaptability of graphene in biological systems, researchers have developed a range of graphene derivatives, collectively known as the graphene family of materials. These include graphene oxide (GO), reduced graphene oxide (rGO), functionalized graphene (FG), and few-layer graphene (FLG), each with distinct structural, surface chemical, and biological properties [53].

#### 4.1.1. Dental Caries

##### Inhibition of Cariogenic Bacteria

Dental caries is a chronic bacterial infectious disease caused by the disruption of the oral microbiome. The core cause is the adhesion of cariogenic bacteria, mainly *S. mutans*, to the tooth surface, forming a dense biofilm. This results in the gradual demineralization and destruction of dental hard tissues due to acid production from sugar metabolism. Traditional treatments for dental caries mostly rely on mechanical removal and filling repairs, with preventive measures dominated by fluoride or antibacterial agents [3]. However, these methods have limited efficacy and potential side effects. Therefore, developing new, safe, and highly targeted anti-caries materials has become a research hotspot in recent years.

Graphene and its derivatives can directly inhibit the proliferation of *S. mutans* and other cariogenic bacteria. The two-dimensional sheet structure of GO and its negatively charged oxygen-containing functional groups allow it to adsorb tightly to bacterial surfaces. Through the “nanoscale blade” effect, GO physically disrupts the integrity of the bacterial cell membrane, inducing cytoplasmic leakage, metabolic disorder, and cell death [12]. In addition, metal-functionalized graphene has also been shown to have significant value in the prevention and treatment of dental caries. A recent study evaluated the antibacterial activity of dental adhesives containing biogenic and chemically synthesized silver nanoparticles (Ag NPs) and silver nano-graphene oxide nanocomposites (Ag@nGO NCs). The results showed that the Ag@nGO NCs group had an antibacterial rate of over 90%, exhibiting the strongest antibacterial effect. No significant differences were observed in dentin bond strength tests among all groups [75]. Moreover, Mao and others have developed a novel graphene oxide-copper composite material that effectively inhibits the growth of *S. mutans*, even altering the biofilm structure, damaging the production and distribution of extracellular polysaccharides, and inhibiting the expression of extracellular polysaccharide-related genes such as gtfB, gtfC, and gbpB, providing a new direction for graphene family applications [76].

Furthermore, aminated graphene oxide (AGO), a functionalized derivative of graphene that has attracted considerable attention in recent years, has also shown outstanding performance in anti-caries research. AGO introduces cationic amino groups into the GO sheets, which not only enhance its stability and biocompatibility in the oral environment but also improve its affinity for bacterial membranes and its bactericidal ability. AGO materials prepared by Lu and others have a triple-action antibacterial effect against *S. mutans*, with the positively charged amino groups on the surface electrostatically targeting adsorption to the negatively charged bacterial surface, enhancing GO’s physical “blade” effect, and exhibiting photothermal therapy (PTT) activity. AGO can rapidly encapsulate *Streptococcus mutans* and destroy its cell membrane structure, leading to morphological distortion, content leakage, and effectively inhibiting biofilm formation and its structural integrity [77].

##### Promoting Remineralization

Dental caries is essentially the imbalance between the dynamic demineralization and remineralization of dental hard tissues in an acidic microenvironment, where the acidic products produced by *S. mutans* and other cariogenic bacteria’s sugar metabolism directly cause enamel and dentin demineralization. Effective caries prevention and control not only require the removal of biofilms and inhibition of bacterial activity but also need to restore the mineralization balance of dental tissues. In recent years, graphene and its derivatives have shown significant potential in promoting remineralization of teeth, with most studies focusing on their role as carriers or synergistic components of inorganic nanomaterials to enhance mineral deposition, improve material mechanical properties, and biological activity.

GO contains functional groups such as carboxyl, hydroxyl, and epoxy groups, which can chemically coordinate with calcium ions, facilitating the deposition of calcium phosphate ions and serving as excellent templates for the formation of mineral products like hydroxyapatite. As a functional interface material, GO is often co-applied with bioactive glass (BAG), and the GO-BAG composite system constructed not only exhibits excellent remineralization ability but also improves bonding strength, material hardness, and anti-demineralization properties, while showing good biocompatibility with odontoblasts [78]. Furthermore, some researchers have synthesized mesoporous bioactive glass nanoparticles (MBN@GOQD) coated with graphene oxide quantum dots (GOQDs), which have good ion release capability and hydrophilicity, accelerating hydroxyapatite formation and efficiently sealing dentinal tubules, showing potential for treating dentin hypersensitivity [79].

Fluorinated graphene oxide (FG) as a novel two-dimensional nanomaterial has shown superior overall performance in enamel remineralization compared to nanohydroxyapatite (n-HAp). Studies have shown that FG not only effectively improves the microhardness of both superficial and deeper (≥100 μm) enamel but also promotes the formation of fluoroapatite, enhancing acid resistance and mineral stability. The two-dimensional sheet structure provides good permeability and calcium–phosphate ion binding ability, further improving remineralization efficiency [80]. At the same time, the fluorine atoms in FG are covalently attached to the carbon skeleton, causing the carbon bonds to transition from the sp^2^ configuration to the sp^3^ configuration. This structural change endows FG with unique optical properties, including full-color emission, which improves the translucency and brightness of enamel, enhancing the aesthetic appearance of demineralized areas [81].

#### 4.1.2. Oral Squamous Cell Carcinoma

Oral squamous cell carcinoma (OSCC) is the most common type of oral malignancy. Its treatment strategies still face challenges such as strong tumor invasiveness, high postoperative recurrence rates, and significant drug resistance. Graphene and its derivatives (graphene-based nanomaterials), including GO, GQDs, and AGO, have shown great potential in the precise treatment of OSCC due to their excellent physicochemical properties (such as ultra-high surface area, ease of functionalization, and good biocompatibility) and multi-modal therapeutic capabilities [82].

##### Drug Delivery Platforms

Oral squamous cell carcinoma (OSCC) is one of the most prevalent oral malignancies worldwide. Although clinical treatment mainly relies on surgical resection combined with radiotherapy and chemotherapy, traditional therapies often lead to various adverse effects, such as xerostomia caused by salivary gland damage, speech disorders, and difficulties in eating. These complications significantly impair the patient’s postoperative quality of life and impose long-term psychological burdens. Therefore, the development of novel drug delivery systems with strong targeting ability, low side effects, and controllable efficacy has become a research hotspot in OSCC treatment [83,84].

Graphene oxide (GO), with its unique two-dimensional sheet-like structure, large specific surface area, and abundant surface functional groups, demonstrates excellent drug-loading capacity and potential for functionalization [53,85]. GO can load various chemotherapeutic agents via π–π stacking, hydrogen bonding, or electrostatic interactions, and can be further chemically modified to enhance targeting capabilities or introduce stimuli-responsive release properties. Studies have shown that GO can serve as a nanocarrier for multiple anticancer drugs, such as doxorubicin, camptothecin, and methotrexate [86]. Among these, GO-DOX nanoplatforms can achieve drug loading capacities up to 200%, significantly higher than traditional nanocarriers [87].

However, pristine GO has limited stability in aqueous environments and tends to aggregate and precipitate, especially after loading hydrophobic drugs. To address this, researchers have introduced hydrophilic polymers, peptides, and cationic polymers to improve its stability and biocompatibility [88]. In recent years, targeted delivery systems for OSCC have undergone continuous optimization. For example, to overcome the electrostatic repulsion between negatively charged GO and nucleic acids, researchers designed a PEI-functionalized GO platform with a positive surface charge, enabling the loading of miRNA inhibitors through electrostatic interactions. This system effectively inhibited OSCC cell migration and proliferation, exhibited excellent cellular uptake and nuclear transport, and significantly suppressed tumor growth in vivo, while upregulating the expression of PTEN and p53 proteins [89]. In another study, a novel graphene quantum dot-based nanocarrier (GPt) was developed for the delivery of cisplatin. PEG modification enhanced its stability and cellular uptake efficiency. This composite material significantly increased drug accumulation at tumor sites, with intratumoral concentrations far exceeding that of free cisplatin. It also exhibited pH-responsive release behavior, effectively releasing Pt in the acidic tumor microenvironment, thereby enhancing OSCC cell apoptosis and S-phase arrest. Especially under hypoxic conditions that activate the HIF-1α signaling pathway and reduce chemotherapy sensitivity, the GPt system demonstrated the ability to reverse drug resistance. In xenograft models, GPt showed superior antitumor efficacy and lower hepatic and renal toxicity compared to free cisplatin, indicating promising potential for OSCC therapy [90].

Furthermore, the gastrin-releasing peptide receptor (GRPR), which is overexpressed in various tumors including head and neck squamous cell carcinoma, provides an ideal target for precision therapy. A targeted GO-based nanoplatform (DOX@NGO-BBN-AF750) was developed to deliver doxorubicin to OSCC. In this system, DOX was loaded onto GO via π–π interactions and hydrogen bonding, and GRPR antagonist peptide BBN was used for tumor targeting. In HSC-3 cells, the nanomaterial was efficiently internalized and showed accelerated drug release under acidic conditions (pH 5.6), reaching a release rate of 19.8% within 24 h. In vitro experiments demonstrated strong antitumor activity and pH responsiveness, suggesting its potential therapeutic value in OSCC treatment [91].

##### Photothermal Therapy (PTT)

Graphene and its derivatives have been widely used in PTT-related research in recent years due to their excellent photothermal conversion capabilities, broad-spectrum NIR absorption, high thermal conductivity, surface modifiability, and good biocompatibility. Researchers have developed an amino-modified graphene oxide (AGO) nanosheet as an efficient photothermal agent for the treatment of oral squamous cell carcinoma (OSCC). This material has a high positive charge (+54 mV), excellent photothermal conversion efficiency, and tumor elimination ability. After intratumoral injection and a single NIR irradiation, AGO can achieve rapid and efficient tumor ablation, with its synergistic cell adhesion and photothermal-induced apoptosis mechanism forming the core of the therapy. After treatment, AGO, along with scab formation, is naturally shed, alleviating the toxic issues caused by long-term material accumulation [92]. Furthermore, researchers developed a dual-targeted, pH/NIR-responsive graphene oxide nanodrug delivery system (GHHD) to enhance the chemo-photothermal combination therapy of OSCC. The system uses GO as a carrier, surface-modified with hyaluronic acid (HA) and HN-1 peptide to target CD44 receptors and OSCC-specific epitopes, and loaded with doxorubicin (DOX). Under acidic conditions (pH 5.0) and 808 nm laser irradiation, the cumulative release of DOX can reach 88.3%. In a mouse xenograft model, GHHD combined with NIR treatment achieved nearly complete tumor elimination without recurrence, and no significant toxicity was observed in the major organs. This study shows that the GO-based dual-targeted nanocarrier system offers significant advantages in precise drug delivery, thermosensitive controlled release, and synergistic efficacy, providing a new nanomedicine strategy for comprehensive OSCC treatment [93].

Additionally, a graphene oxide-based lipid modulation nanoplatform (NSD) was developed, which co-delivers ACLY inhibitor SB-204990 and DOX to achieve a tri-modal synergy of lipid starvation, chemotherapy, and photothermal therapy. Under acidic conditions and 808 nm laser irradiation, NSD accelerates drug release, with DOX and SB-204990 release rates reaching 83.3% and 79.1%, respectively, within 72 h. In vitro experiments show that NSD + laser treatment reduces the survival rate of OSCC cells to about 20%, with late-stage apoptosis significantly rising to 33.2%. In animal experiments, the NSD + laser group exhibited a 99.4% tumor inhibition rate, with some mice achieving complete tumor eradication, and no obvious organ toxicity was observed. This study demonstrates the advantages of lipid metabolism intervention and photothermal synergy, providing an efficient and safe multi-modal treatment strategy for OSCC [94].

##### Photodynamic Therapy (PDT)

In addition to drug delivery and photothermal therapy, another promising therapeutic modality for OSCC is photodynamic therapy (PDT). Unlike PTT, which relies on hyperthermia to ablate tumor tissues, PDT employs light-activated photosensitizers to generate cytotoxic reactive oxygen species (ROS), thereby inducing apoptosis or necrosis in tumor cells. Graphene-based nanomaterials not only serve as efficient carriers for chemotherapeutics but also function as platforms for light-mediated therapies.

Photodynamic Therapy (PDT) is an emerging non-invasive tumor treatment strategy that induces apoptosis or necrosis in tumor cells by activating photosensitizers (PS) to generate Reactive Oxygen Species (ROS). This technique relies on three key elements: photosensitizer, specific wavelength light, and oxygen in tissues. After the photosensitizer accumulates at the tumor site, it is excited by visible or near-infrared (NIR) light, transitioning to an excited state. As it returns to the ground state, energy is transferred to oxygen molecules, producing singlet oxygen (^1^O_2_) and other ROS. These ROS then damage the cancer cell membranes, mitochondria, or DNA, inducing cell death [95]. Compared to traditional chemotherapy, PDT offers advantages such as precise localization, low toxicity, and a low risk of inducing resistance. It is especially suitable for treating local malignant tumors in the head and neck.

In recent years, graphene and its derivatives have shown unique advantages in the field of PDT due to their excellent drug-loading capacity, optical properties, and potential for biological functionalization. On one hand, GO and GQDs have excellent optical absorption properties and π–π stacking structures, allowing them to efficiently load common photosensitizers like Ce6, Methylene Blue, and ICG. This improves their water solubility and biological stability, reducing in vivo self-aggregation and photobleaching phenomena [96]. On the other hand, the rich surface functional groups of GO facilitate the binding with targeting ligands (such as GRPR antagonistic peptides, folic acid, RGD peptides, etc.), enabling targeted delivery to tumor tissues and enhancing the specificity and accumulation efficiency of PDT [97]. Additionally, graphene quantum dots (GQDs) themselves can also serve as photosensitizers in the PDT process. Notably, a recent study synthesized PEG-modified GQDs (GQD-PEG) and applied them to PDT for OSCC. This material showed excellent aqueous stability, biocompatibility, significant generation of ROS under 560 nm laser irradiation, and tumor-targeting capacity. In vivo and in vitro experiments demonstrated that GQD-PEG effectively inhibited tumor growth under light exposure, while also activating the host immune response, inducing CD8^+^ T cell tumor infiltration, and promoting the release of inflammatory cytokines such as IFN-γ and TNF-α, thereby achieving a synergistic effect of PDT and tumor immunotherapy. This research provides strong support for the immune combination strategy of GQD-based photosensitizer platforms in OSCC treatment [98].

In recent years, Nitrogen-doped Graphene Oxide Dots (NGODs) have been developed as new photosensitizers for PDT in oral cancer due to their good photostability, biocompatibility, and photocatalytic performance. Research has shown that by introducing ascorbic acid (AA) as a hole scavenger, NGODs’ H_2_O_2_ production under visible light can be effectively enhanced, achieving Type I ROS generation mechanisms under hypoxic conditions. This overcomes the traditional limitation of PDT’s strong dependence on oxygen. Specifically, AA reacts with photogenerated holes, inhibiting electron-hole recombination, thereby promoting electron reactions with oxygen molecules to generate H_2_O_2_. This increases ROS production and enhances cytotoxicity. Additionally, the incorporation of AA also inhibits the triplet energy transfer process, reducing the generation of ^1^O_2_ and shifting the reaction towards the Type I mechanism, which is more suitable for the hypoxic environment of solid tumors. The system demonstrated significant cytotoxicity against various cancer cell lines such as PC-9, HONE-1, HCT-116, and OECM-1, while having minimal effects on normal fibroblasts and oral keratinocytes, exhibiting good tumor selectivity [99].

#### 4.1.3. Periodontitis

Periodontitis is a chronic inflammatory disease triggered by bacterial infection and accompanied by host immune imbalance. It is characterized by gum tissue destruction, alveolar bone resorption, and tooth mobility, which can eventually lead to tooth loss. Traditional treatment relies on mechanical debridement and antibiotic interventions; however, the frequent emergence of multidrug-resistant bacterial strains and the risk of microbial re-infection significantly limit its efficacy. Therefore, the development of new treatment methods that combine antibacterial, anti-inflammatory, and tissue repair functions has become a research hotspot. Graphene and its derivatives, due to their excellent physicochemical properties and biological functions, have shown broad application potential in the treatment of periodontitis.

##### Inhibition of Periodontal Pathogens

Bacteria are widely considered to be the initiating factor in the development of periodontitis, with *P. gingivalis* playing a key role. In a healthy state, the microbial community in the periodontal tissues maintains a dynamic balance with the host immune system, resulting in a mild but effective immune response that prevents excessive microbial proliferation [100]. However, when this balance is disrupted, plaque biofilm begins to accumulate on the tooth surface, with pathogens such as *P. gingivalis* gradually dominating. These pathogens form the “red complex” in cooperation with other species such as Fusobacterium nucleatum and Treponema denticola, which collectively trigger the development of periodontitis [101]. Although *P. gingivalis* is not the most abundant pathogen in the biofilm, it has a significant “microbiota-driving” characteristic. It can produce a variety of virulence factors, such as lipopolysaccharides, gingipains, and capsular polysaccharides, to manipulate the host immune response, weaken phagocyte function, and interfere with Toll-like receptor (TLR2/TLR4) signaling pathways [102]. This allows it to escape immune clearance and induce a chronic inflammatory response. This immune modulation not only disrupts the existing microbiota homeostasis but also creates a suitable environment for other facultative or anaerobic pathogens, promoting periodontal dysbiosis and the persistence of chronic inflammation.

In recent years, the potential of graphene and its derivatives in inhibiting periodontal pathogens has gained widespread attention. Research has shown that GO at 40 μg/mL can exhibit 99% antibacterial activity against *P. gingivalis* and also significantly inhibit *F. nucleatum* [103]. Furthermore, the non-specific mechanism of GO reduces the risk of inducing resistance, demonstrating its broad application potential as a new antibacterial material in the dental field [104].

Additionally, researchers have developed a targeted antibacterial photodynamic therapy (aPDT) system based on DNA aptamer-modified nanoscale graphene oxide (DNA-aptamer-NGO), which can specifically recognize and kill *P. gingivalis*. This system generates ROS under 980 nm laser irradiation, significantly inhibiting bacterial growth, destroying the biofilm, and reducing metabolic activity. At the same time, it regulates the expression of virulence-related genes (such as fimA and rgpA) and exhibits good biocompatibility, demonstrating its potential as a targeted diagnostic and therapeutic platform for periodontal pathogens [105].

##### Promotion of Periodontal Bone Regeneration

The tissue damage caused by periodontitis not only involves bacterial infection but is also accompanied by host immune imbalance and a decline in the regenerative capacity of periodontal tissues [106]. Graphene and its derivatives, with their tunable surface chemistry, excellent mechanical properties, and good biological activity, have shown great potential in the field of periodontal tissue engineering in recent years, particularly in promoting stem cell regeneration, regulating immune responses, and restoring alveolar bone structure.

GO has been proven to significantly promote the osteogenic differentiation of periodontal ligament stem cells (PDLSCs). Related studies have shown that poly(ε-caprolactone) (PCL) scaffolds coated with GO improve material hydrophilicity and cell adhesion ability, while enhancing the proliferation and mineralization capacity of PDLSCs, showing significant potential in constructing active scaffolds and promoting alveolar bone repair [107].

A study developed a GO-coated collagen scaffold for repairing class II furcation defects in dogs. The scaffold showed good biocompatibility, high mechanical strength, and enhanced osteoblast adhesion. It promoted bone regeneration, neovascularization, and immune modulation in rat and canine models, with superior repair effects compared to traditional collagen scaffolds. This was due to enhanced protein adsorption, fibroblast and M2 macrophage recruitment, and accelerated tissue regeneration, demonstrating its potential in periodontal tissue engineering [108].

**Figure 3:**
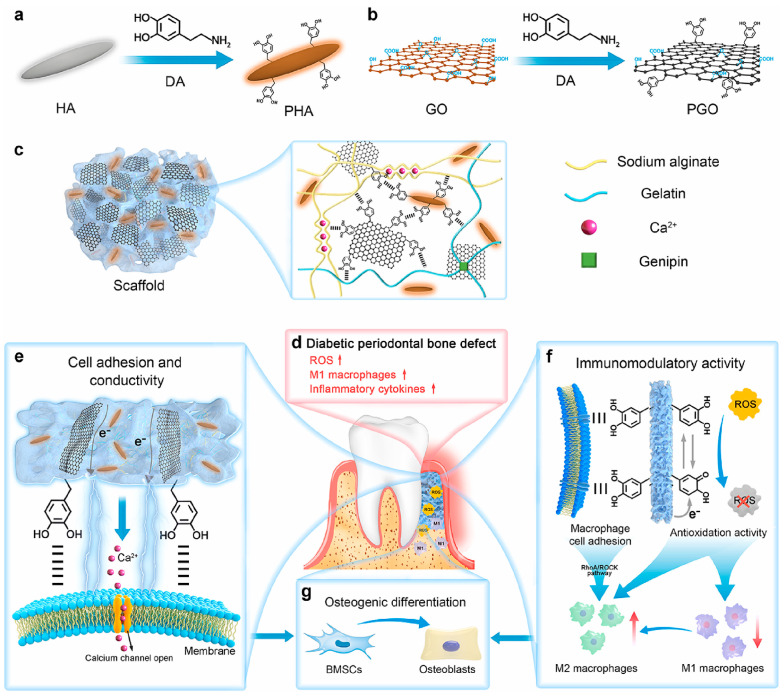
Multifunctional PGO-PHA-AG scaffold for diabetic periodontal bone regeneration. (**a**) Synthesis of PHA; (**b**) Synthesis of PGO; (**c**) Dual crosslinked scaffold network; (**d**) Diabetic inflammatory microenvironment; (**e**) Activation of Ca^2+^ channels through endogenous electrical signals; (**f**) PDA-mediated ROS scavenging and immunomodulation; (**g**) Synergistic effects of conductivity and immune regulation promote bone regeneration. Reprinted with permission from [109], Copyright 2022, Elsevier.

In addition to promoting cell differentiation, GO-based materials also have good immune regulatory capabilities. Li et al. constructed a PGO-PHA-AG composite scaffold, where dopamine-reduced graphene oxide (PGO) endowed the material with excellent conductivity, which can activate Ca^2+^ channels to promote osteogenesis and act synergistically with mechanisms such as macrophage polarization and ROS scavenging. As shown in Figure 3, the scaffold significantly scavenged reactive oxygen species (ROS) in a high-glucose inflammatory microenvironment, with a DPPH free radical scavenging rate of 93%. Meanwhile, the scaffold in a diabetic periodontal bone defect model activated Ca^2+^ channels to transmit endogenous electrical signals, regulating macrophage glycolysis and the RhoA/ROCK pathway, promoting M1 to M2 polarization, and enhancing osteogenic factor expression, thus achieving excellent bone regeneration effects [109].

#### 4.1.4. Peri-Implantitis

##### Inhibiting Bacterial Adhesion

Peri-implantitis is an inflammatory disease occurring around dental implants, primarily induced by bacterial biofilms. It is characterized by inflammatory reactions in the surrounding soft tissues accompanied by progressive marginal bone resorption. Chronic inflammation leads to excessive activation of osteoclasts and disruption of the bone remodeling balance, ultimately resulting in bone loss and implant failure. As implant coating materials, graphene and its derivatives have shown great potentials in inhibiting bacterial adhesion due to their unique physicochemical properties, antibacterial characteristics, and surface modification abilities. Firstly, graphene coatings significantly reduce the adhesion of pathogenic bacteria to implant surfaces. Agarwalla et al. reported that vacuum-assisted deposition of graphene nano-coatings could long-term inhibit biofilm formation and hyphal development of Candida albicans on implant surfaces without relying on antifungal drugs. The material lowers surface free energy and roughness, enhancing hydrophobicity, which significantly hinders initial microbial attachment without inducing resistance. Studies have shown that just two layers of graphene transfer are enough to form a durable antibacterial barrier, providing a new approach to preventing fungal-related peri-implantitis [110]. Moreover, graphene materials exhibit broad-spectrum antibacterial activity against various typical oral pathogens. Wei et al. developed Ti-0.125G (graphene-enhanced titanium) materials that significantly reduced the biofilm volume of *Porphyromonas gingivalis*, *Fusobacterium nucleatum*, and *Streptococcus mutans*. The inhibition of *P. gingivalis* was most prominent within 96 h, and its antibacterial mechanism was attributed to the good conductivity imparted by graphene, which enabled the Ti-0.125G surface to capture electrons, interfering with bacterial respiration and blocking ATP generation, thereby inducing bacterial death. At the same time, graphene, under high-temperature sintering, forms TiC with titanium, enhancing its antibacterial effect [111].

In addition, the abundant oxygen functional groups on the GO surface not only give it a negative charge to repel bacteria but also enhance its ability to bind with drugs, providing potential for synergistic antibacterial effects. Liu et al. successfully coated minocycline and GO on ultra-fine titanium surfaces, effectively blocking initial staphylococcal attachment and inhibiting mature biofilm formation while maintaining good biocompatibility [112]. Notably, GO coatings exhibit stability and durability in biofilm inhibition. Rosa et al. demonstrated that graphene coatings made by chemical vapor deposition retained >98% coverage after pig jawbone implantation, plaque exposure, and washing treatments, withstanding bio-corrosion without reducing antibacterial performance [113].

##### Promoting Osseointegration and Soft Tissue Sealing

Osseointegration refers to the direct and firm structural connection between the implant and surrounding bone tissue, where no connective tissue is observed under optical microscopy. This process typically involves initial blood clotting, osteoblast adhesion and proliferation, matrix secretion and calcification, and later bone remodeling, ultimately forming a stable “bone-implant interface,” which is essential for implant success. In addition to excellent antibacterial properties, graphene and its derivatives show significant advantages in improving the biological activity of implant surfaces, promoting osseointegration, and enhancing soft tissue sealing, providing important material support for improving long-term implant stability [114,115].

To promote this biological process, graphene materials significantly enhance osseointegration by improving cell adhesion, enhancing osteogenic signaling, and regulating the microenvironment. Lu et al. developed a graphene-coated titanium material that showed stronger growth factor adsorption capability and promoted the expression of bone formation-related genes and proteins in bone marrow mesenchymal stem cells (BMSCs) in the presence of autologous concentrated growth factors (CGFs). This was achieved by activating the RhoA/ROCK1/ERK1/2 cell cytoskeleton-related signaling pathways, accelerating their differentiation into osteoblasts [116]. Kang et al. applied meniscus-dragging deposition (MDD) technology to evenly deposit reduced graphene oxide (rGO) on the titanium surface, significantly improving its hydrophilicity and surface energy while reducing surface roughness and enhancing cell adaptability. On this basis, hMSCs showed significantly enhanced adhesion and spreading on the rGO-Ti surface, and both ALP activity and calcium nodule formation were significantly higher in the osteogenic early and late stages compared to the uncoated group. Mechanistically, the abundant defect structure and residual oxygen groups on the rGO surface help protein adsorption and activation of cell signaling pathways, coordinating cell–matrix interactions, thereby enhancing osteogenesis induction [117]. Shin et al. further coated rGO on SLA-treated titanium surfaces, and rGO coatings not only improved implant surface physicochemical properties but also significantly induced osteogenic differentiation of hMSCs without exogenous osteogenic factors, achieving excellent osseointegration in animal experiments, superior to traditional biomolecular modification strategies like rhBMP-2, with good biocompatibility and clinical translation potential [118].

Additionally, the excellent photothermal conversion ability of graphene has been applied to synergistically promote bone regeneration. Yang et al. developed a TiO_2_-GO photothermal composite structure that, under near-infrared (NIR) light, not only enhanced antibacterial activity but also induced osteoblasts (MC3T3-E1) to upregulate osteogenic genes such as OPN, OCN, and BSP, significantly promoting early osseointegration of the implants, even under non-infection conditions, and showing good osseointegration ability [119].

In terms of soft tissue, good gingival sealing is key to the long-term stability of implants. Wei et al. constructed a graphene-enhanced titanium material (Ti-0.125G) using spark plasma sintering, which significantly promoted the adhesion, proliferation, and migration of gingival fibroblasts by optimizing surface hydrophilicity and mechanical properties. It also activated FAK-related signaling pathways and ECM protein expression, enhancing cell integration on the material surface. In multi-species co-culture models, Ti-0.125G effectively inhibited bacterial biofilm formation, maintained fibroblast dominance, and significantly improved soft tissue sealing around the implant, helping prevent peri-implant infection [111]. Gao et al. developed a “sandwich structure” implant abutment coating by embedding GO beneath a mineralized collagen layer, achieving remote controllable antibacterial activity while significantly enhancing soft tissue sealing performance. The outer mineralized collagen not only provided good biocompatibility but also enhanced the adhesion of gingival fibroblasts, promoted the formation of F-actin stress fibers, and improved the expression of adhesion proteins (vinculin and integrin β1), establishing a stable cell–substrate connection [120]. Additionally, the dispersed hydroxyapatite crystals and surface hydrophilicity further improved cell morphology and spreading, jointly promoting soft tissue attachment and barrier function reconstruction in infected environments [116]. The typical applications of graphene and its derivatives in oral diseases are summarized in Table 1.

### 4.2. Black Phosphorus Nanosheets (BPNSs)

Black phosphorus (BP) is a layered elemental material that can be fabricated into two-dimensional black phosphorus nanosheets (BPNSs) through approaches such as mechanical or liquid-phase exfoliation and chemical vapor deposition [121]. With a tunable bandgap ranging from 0.3 to 1.7 eV, BP exhibits strong light absorption and anisotropic electrical properties, enabling broad-spectrum responsiveness and excellent potential in photothermal (PTT) and photodynamic (PDT) therapies [122]. As a result, BP exhibits remarkable potential in photothermal therapy (PTT) and photodynamic therapy (PDT), and is considered one of the most promising emerging two-dimensional photosensitive materials. Unlike graphene, BP is composed of phosphorus, an essential mineral element. Its degradation products are physiologically compatible phosphates (PO_4_^3−^), which not only prevent long-term accumulation but also participate in bone regeneration, thereby ensuring both safety and therapeutic efficacy [123]. Overall, BP offers promising applications in antibacterial therapy, cancer treatment, and tissue regeneration.

#### 4.2.1. Dental Caries

##### Inhibition of Cariogenic Bacteria

As a novel two-dimensional material, BP exhibits significant potential in antibacterial and anti-caries research due to its unique physical structure and electronic properties. Similarly to other 2D nanomaterials such as graphene, BP possesses a sharp lamellar structure and high specific surface area, allowing it to directly contact and physically disrupt bacterial membranes, leading to cytoplasmic leakage, metabolic imbalance, and cell death—an effect described as a “nano-blade” antibacterial mechanism [124]. Additionally, the presence of abundant lone-pair electrons on its surface facilitates oxygen adsorption. In the moist and oxygen-rich oral environment, BP can continuously generate reactive oxygen species (ROS), thereby activating bacterial oxidative stress pathways and inducing fatal damage such as lipid peroxidation and DNA strand breakage [125].

Moreover, BP demonstrates excellent photothermal conversion under near-infrared (NIR) irradiation [126]. This photothermal-assisted antibacterial effect has been confirmed to enhance bacterial inactivation and improve the spatial-temporal controllability of treatment [127]. In one study, BP/Au nanocomposites exhibited significant temperature elevation and synergistic bactericidal activity under 808 nm NIR light, effectively inhibiting *E. faecalis* growth and disrupting its biofilm structure [128].

Recently, Ran et al. developed a mussel-inspired hydrogel (BP@CP5) loaded with BPNSs, which integrates wet adhesion, photothermal antibacterial activity, and in situ remineralization for precise intervention in early caries. Composed of catechol-modified chitosan and thermosensitive PLGA-PEG-PLGA, the hydrogel demonstrates excellent injectability and temperature-sensitive gelation, adhering stably to tooth surfaces for over 24 h. Under NIR irradiation, the embedded BPNs rapidly increase in temperature to kill *S. mutans* and *S. salivarius*, significantly suppressing caries progression in animal models with good biocompatibility. This highlights the promising application of BPN-loaded hydrogels in non-invasive caries therapy [129].

##### Promotion of Remineralization

In recent years, the development of novel nanomaterials with dual antibacterial and remineralization capabilities has become a focus in caries treatment. In oral fluids, BP can gradually degrade into inorganic ions such as phosphate (PO_4_^3−^) and phosphite (HPO_3_^2−^), which can bind with Ca^2+^ in saliva to induce local hydroxyapatite (HA) crystal precipitation, thereby promoting remineralization of demineralized regions [129]. Additionally, the abundant active sites on BP surfaces provide high-affinity binding platforms for Ca^2+^, significantly enhancing local calcium ion enrichment and accelerating HA nucleation and growth. Compared to conventional calcium–phosphate remineralization systems, BP not only serves as a sustainable phosphorus source but also acts as a mineralization template due to its 2D layered structure, guiding the transformation of amorphous calcium phosphate (ACP) into ordered HA crystals. This results in denser and mechanically stronger newly formed mineral layers [42].

**Figure 4:**
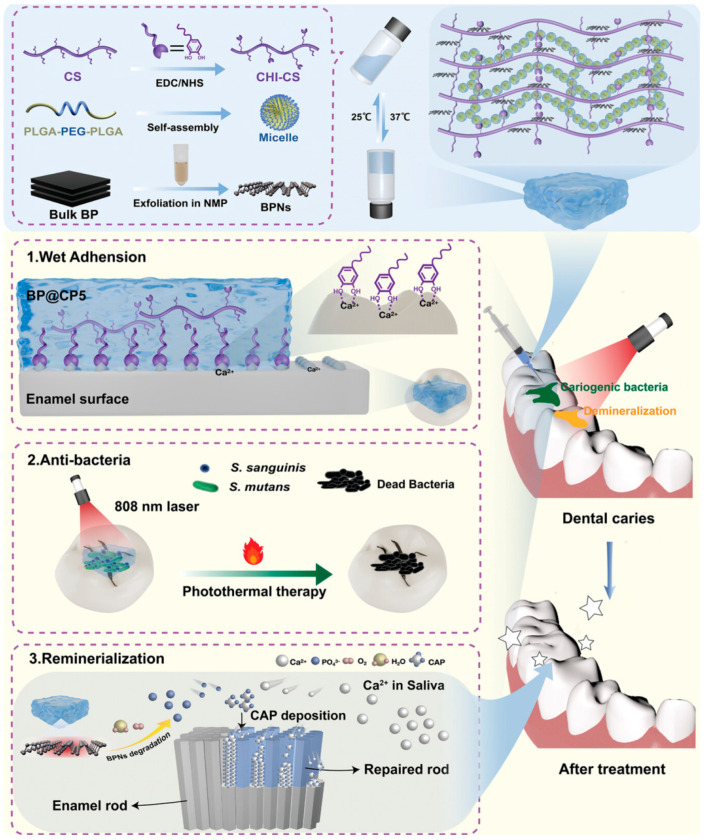
Schematic illustration of the preparation of the BP@CP5 hydrogel and its therapeutic mechanism to control early caries. (1) Wet adhesion properties. (2) BPNs-mediated PTT against cariogenic bacteria. (3) Remineralization based on BPNs degradation [129]. Copyright 2024, Wiley.

As shown in Figure 4, in the aforementioned BP@CP5 hydrogel system, researchers further observed that the material could continuously release PO_4_^3−^ ions under humid conditions and induce the formation of a dense and continuous hydroxyapatite-like layer on the enamel surface. Experimental data showed that treatment with this hydrogel resulted in the formation of a remineralized layer up to 15 μm thick on acid-etched enamel, with a Ca/P ratio close to that of natural enamel and significantly higher microhardness compared to commercial agents, fully demonstrating its potential and advantages in remineralization-based repair [129].

#### 4.2.2. Oral Squamous Cell Carcinoma

Recent studies have revealed that BP not only performs well in photothermal therapy (PTT) and photodynamic therapy (PDT), but also possesses unique intrinsic anti-tumor activity. It can selectively induce tumor cell death even without external stimulation. This anticancer effect stems from BP’s distinct physicochemical properties and its responsive degradation behavior in the tumor microenvironment. In the oxidative stress conditions of cancer cells, which are rich in reactive oxygen species (ROS), BP readily degrades and releases large amounts of phosphate ions (PO_4_^3−^). These degradation products are biocompatible and buffering in nature, and may disrupt cellular metabolic balance to exert anti-tumor effects [130].

On one hand, phosphate ions accelerate intracellular ATP hydrolysis, rapidly depleting the energy reserves of cancer cells, increasing the AMP/ATP ratio, and activating the energy-sensing AMPK pathway. Prolonged AMPK activation suppresses downstream signaling such as mTOR, which is involved in metabolism and proliferation, thereby promoting energy-depletion-induced apoptosis [131]. On the other hand, the high phosphate concentration from BP degradation can damage mitochondrial membrane potential, trigger cytochrome C release, and activate the caspase cascade, initiating endogenous apoptotic pathways. Studies also suggest that this process inhibits superoxide dismutase (SOD) activity, enhances lipid peroxidation, and further increases ROS accumulation, aggravating oxidative damage [132].

Li et al. developed a pH-responsive charge-reversal nanoplatform (BP@PDA-PAH-DMMA) based on BP nanosheets. By coating the surface with polydopamine (PDA), the material’s stability and photothermal properties were enhanced. Additionally, poly(allylamine hydrochloride) (PAH) and dimethylmaleic anhydride (DMMA) were grafted onto the surface, allowing the nanoplatform to reverse its surface charge from negative to positive under acidic tumor conditions. This significantly improved tumor cell uptake and targeted accumulation at tumor sites. In oral cancer cell lines CAL-27 and SAS, the platform exhibited strong photothermal killing ability under 808 nm NIR laser irradiation, reducing cell viability to below 10% at a concentration of 100 μg/mL. In a mouse oral cancer xenograft model, local tumor temperature rapidly increased to approximately 54.9 °C after injection, sufficient to induce tumor cell apoptosis, while sparing surrounding normal tissues. This strategy demonstrates excellent photothermal therapeutic effects and biosafety both in vitro and in vivo, offering a promising, safe, and targeted approach for non-surgical treatment of oral cancer [16].

To address the clinical challenge of postoperative tumor recurrence and infection, Li and colleagues designed a “plug-and-play” hydrogel system (BP-Ag@HA-DA-Plu), where BP–silver nanocomposites were embedded in a dopamine-modified hyaluronic acid and thermosensitive Pluronic^®^ F127 hydrogel. This system integrated photothermal therapy with antibacterial and anti-infective capabilities. In vitro experiments showed that after NIR treatment, the survival rate of CT26 colon cancer cells dropped to 13.7%, significantly lower than in the untreated (84.6%) and BP-only (42.9%) groups. Live/dead staining showed minimal surviving cells, confirming strong photothermal cytotoxicity. The embedded silver nanoparticles provided lasting antibacterial and partial tumor-inhibitory effects, making this hydrogel a multifunctional therapy for post-surgical tumor suppression and wound healing [133].

In addition, treatment of oral squamous cell carcinoma may require partial mandibulectomy. The repair of defective bone tissue is crucial for improving postoperative quality of life. Yang et al. applied 3D-printed bioactive glass scaffolds integrated with BP nanosheets for osteosarcoma therapy, validating the photothermal anti-tumor activity of BP and showing that it promoted in situ bone regeneration under physiological conditions—highly significant for postoperative repair [134].

#### 4.2.3. Periodontitis

BP not only possesses excellent biocompatibility and biodegradability—degrading into physiological phosphates that can participate in mineralization—but more importantly, it demonstrates outstanding reactive oxygen species (ROS) scavenging, photo-responsiveness, and immunomodulatory capabilities under inflammatory conditions. These features make BP an ideal intervention material for periodontitis, a disease characterized by intertwined factors of infection, inflammation, and tissue destruction [135].

Diabetic periodontitis is characterized by more severe inflammation, greater tissue damage, and more difficult treatment outcomes, primarily due to the synergistic effects of macrophage polarization imbalance and iron metabolism disorders induced by hyperglycemia. Under such complex metabolic conditions, BP has shown remarkable regulatory effects on immunity and iron homeostasis. Qin et al. designed a CH-BPNs-NBP hydrogel system that integrates the antioxidant capacity of BP nanosheets (BPNs) with the vasodilatory function of NBP. This system effectively corrected iron overload and skewed M1 macrophage polarization under hyperglycemic conditions, restored the expression of iron metabolism-related proteins (TfR1, FPN1, GPX4), and promoted M2 polarization, thereby reducing alveolar bone resorption and highlighting BP’s potential in local immune modulation under systemic disease contexts [136]. Moreover, BP protects the periodontal microcirculation system. Wang et al. confirmed that CH-BPNs-NBP enables synergistic treatment of periodontitis through controlled release of BPNs and NBP. In a rat periodontitis model, this formulation significantly reduced inflammatory cytokines IL-1β and TNF-α by about 65% and 60%, respectively, and increased M2 macrophage marker Arg-1 expression by approximately threefold. Additionally, lymphatic vessel density increased 2.4 times, indicating improved tissue perfusion. Micro-CT results showed a 45% increase in bone volume fraction (BV/TV), suggesting enhanced bone regeneration [137].

BP has also been applied to optimize traditional photodynamic therapy (PDT). Li et al. constructed a BPNS-based system combining indocyanine green (ICG) and aPDT, which effectively addresses oxidative side effects caused by ROS generation in conventional PDT. The BP nanosheets not only boost antibacterial efficiency via ROS but also neutralize excess ROS due to their intrinsic antioxidant properties, maintaining inflammatory balance and protecting surrounding tissue [138]. To tackle immune imbalance caused by persistent neutrophil extracellular traps (NETs) in chronic infections, Tao et al. developed a dual-responsive nanocapsule (E-TA-BP@D) that releases DNase I in response to NIR light and NET microenvironments, clearing NETs while leveraging BP’s photothermal/photodynamic antibacterial effects and osteoinductive properties. The system reshapes the immune microenvironment, promotes M2 macrophage polarization, and supports osteogenic differentiation of bone marrow mesenchymal stem cells (BMSCs). In both periodontitis and apical periodontitis models, E-TA-BP@D significantly suppressed inflammation and promoted bone regeneration, proposing a new therapeutic strategy targeting NET-induced immune responses for chronic infectious bone destruction [139].

The layered structure and large specific surface area of BP facilitate its physical adsorption and intercalation of drug molecules. The presence of numerous unpaired electrons on its surface allows for chemical adsorption or weak bonding with hydroxyl, carboxyl, and amino groups in drugs, improving drug loading capacity and stability [140]. Song et al. developed light-responsive microparticles (MPs) inspired by abalone suckers for localized periodontal drug delivery. These microparticles, fabricated via microfluidic electrospray using alginate and PEGDA to form concave discs, demonstrated enhanced underwater adhesion in the gingival sulcus. Incorporated BP released phosphate in vivo for buffering and bone modulation. Upon NIR irradiation, local temperature increased, enhancing minocycline release and achieving controlled antibacterial therapy [141].

To further enhance tissue regeneration, He et al. designed a BPQDs-modified ADSC system that enhanced bone regeneration and immune modulation in periodontitis. BPQDs promoted ADSC osteogenic differentiation via Wnt/β-catenin and BMP2 pathways, while also inducing M2 macrophage polarization. Inflammatory models showed improved BMSC activity and bone regeneration, with superior therapeutic outcomes compared to ADSCs alone [142].

### 4.3. 2D Metal–Organic Frameworks (2D MOFs)

Two-dimensional metal–organic frameworks (2D MOFs) are nanometer-thick porous materials derived from conventional 3D MOFs, offering larger surface area, more exposed active sites, and improved dispersibility and interfacial adaptability [143]. Their ultrathin yet laterally extended structures allow the formation of stable adhesion layers under dynamic oral conditions, ensuring sustained biological activity. Well-defined pores and tunable ligands provide versatility for drug loading, stimulus-responsive release, and surface functionalization, enabling multifunctional therapeutic platforms [144]. In addition, some 2D MOFs possess excellent charge and proton transport properties, which enhance photothermal conversion, photodynamic therapy, and ROS catalytic efficiency. Together, these advantages highlight the broad potential of 2D MOFs in antibacterial therapy, photo-responsive treatment, and tissue regeneration.

#### Periodontitis

In recent years, the application of two-dimensional metal–organic frameworks (2D MOFs) in the treatment of periodontitis has attracted increasing attention. Benefiting from their multiple therapeutic mechanisms and structural advantages, 2D MOFs demonstrate promising potential for comprehensive intervention. Firstly, the metal nodes in MOFs (such as Cu, Zn, and Fe) can continuously release bactericidal metal ions under physiological conditions, disrupting bacterial membranes, inhibiting enzymatic activity, and interfering with DNA replication, thereby achieving broad-spectrum antibacterial effects against periodontal pathogens [145]. In addition, 2D MOFs incorporating photosensitive ligands such as porphyrins possess excellent photoresponsiveness; upon near-infrared (NIR) irradiation, they can generate photothermal or photodynamic effects that penetrate and disrupt the deeper layers of biofilms, significantly improving treatment efficacy [14]. Moreover, the ultrathin, sharp-edged structure of 2D MOFs exhibits a “nano-knife” effect, which physically tears bacterial membranes and accelerates cell lysis.

**Figure 5:**
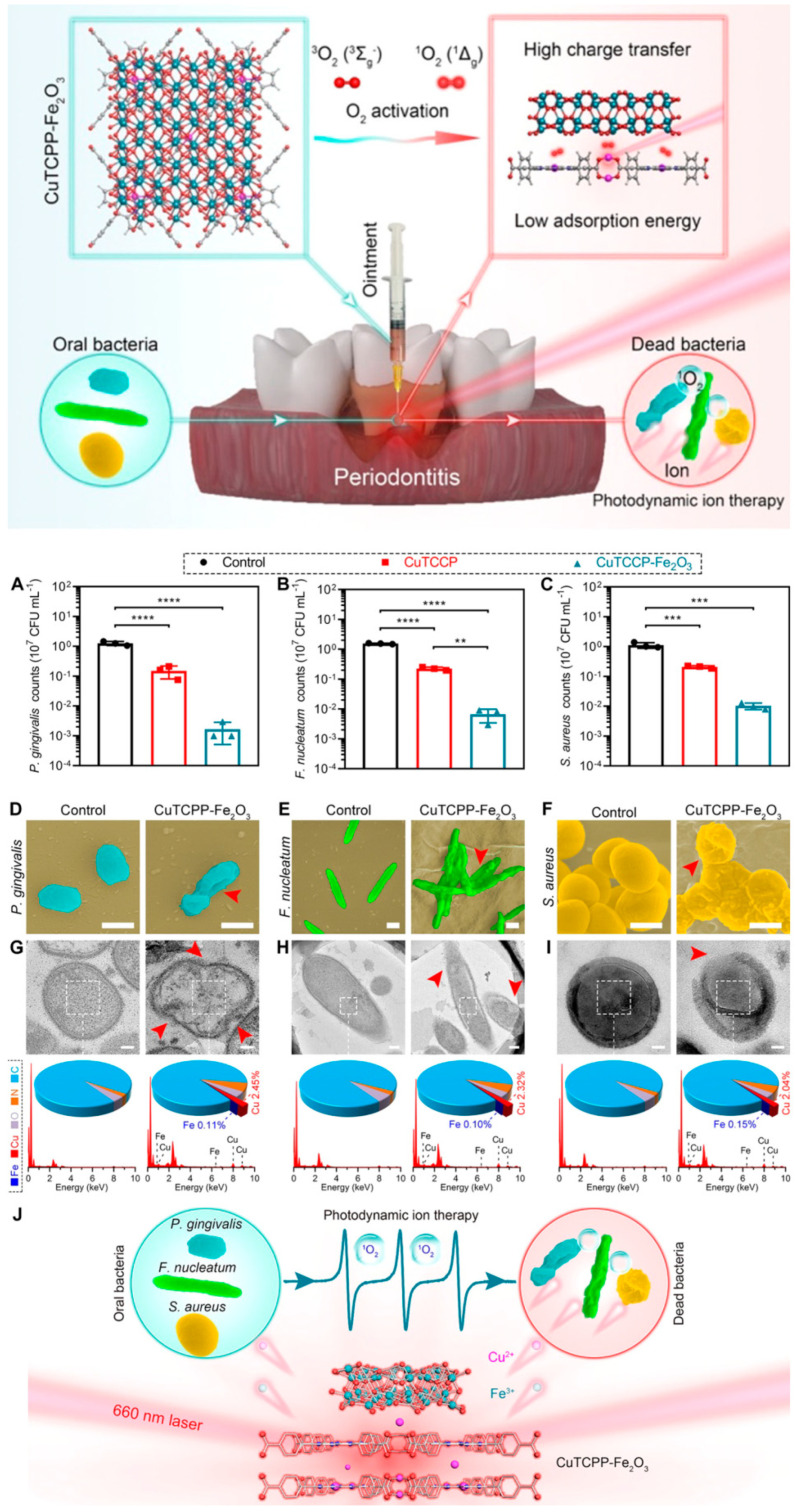
Antibacterial mechanism of photodynamic ion therapy. Viability of *P. gingivalis* (**A**), *F. nucleatum* (**B**), and *S. aureus* (**C**) after treatment with CuTCPP and CuTCPP-Fe_2_O_3_ under 660 nm laser irradiation for 20 min followed by 2 h in the dark. SEM images of *P. gingivalis* (**D**), *F. nucleatum* (**E**), and *S. aureus* (**F**) treated with CuTCPP-Fe_2_O_3_ under the same conditions, showing obvious bacterial membrane disruption. (**G**) TEM image and EDS analysis of *P. gingivalis,* indicating material uptake and structural destruction. (**H**) TEM image and EDS analysis of *F. nucleatum*, revealing Cu and Fe internalization and cell morphology damage. (**I**) TEM image and EDS analysis of *S. aureus*, confirming material penetration and severe membrane disruption. (**J**) Schematic illustration of the antibacterial mechanism: the synergistic action of ROS generation and released ions leads to extensive bacterial membrane damage and eventual cell death. Red arrows indicate the significantly damaged bacteria. Statistical differences: ** *p* < 0.01, *** *p* < 0.001, **** *p* < 0.0001 [14]. Copyright 2021, American Chemical Society.

More importantly, MOFs can be functionalized through the design of organic ligands to achieve multimodal synergistic therapy. This includes the incorporation of anti-inflammatory groups, osteoinductive molecules, or immunoregulatory modules, enabling targeted intervention within the complex inflammatory microenvironment of periodontitis [146]. For example, as shown in Figure 5, Li et al. developed a Fe_2_O_3_-modified porphyrin-based 2D MOF ointment that, under NIR irradiation, synergistically generated ROS to kill periodontal pathogens and disrupt biofilms. It reduced TNF-α and IL-1β levels, promoted M2 macrophage polarization, and activated Wnt/β-catenin signaling to enhance osteogenesis. Animal studies confirmed its combined antibacterial, anti-inflammatory, and regenerative effects in periodontal lesions [14].

However, the current research on 2D MOFs for oral applications remains in its infancy and faces several technical challenges. Due to the complex crystalline structure composed of metal nodes and organic ligands, the controllable synthesis of 2D MOFs remains technically demanding. Common fabrication strategies such as liquid-phase exfoliation, confined growth, and template-assisted synthesis often suffer from low yield, uneven layer thickness, and poor crystallinity [147]. Moreover, some 2D MOF structures exhibit limited stability in aqueous environments, making them less suitable for long-term performance in the saliva-rich and enzyme-active oral cavity. In addition, systematic evaluations of their long-term biosafety, immunocompatibility, and degradation pathways in vivo are still lacking, which hinders their clinical translation. Therefore, future research should focus on developing green and efficient synthesis strategies, enhancing structural stability, and enabling programmable design of biofunctional modules to advance the scalable application and precision therapeutic potential of 2D MOFs in periodontitis treatment [148].

### 4.4. MXene

MXenes are a family of two-dimensional layered materials composed of transition metals and carbon or nitrogen, with a general formula of M_n+1_X_n_T_x_, where M represents an early transition metal (e.g., Ti, Nb, V), X is carbon and/or nitrogen, and T_x_ denotes surface functional groups such as –OH, –F, and =O. Since their initial discovery in 2011 at Drexel University via selective etching of the A-layer from MAX phase precursors (M_n+1_AX_n_), MXenes have emerged as a prominent class of materials with tunable surface chemistry and multi-functional interfaces [149].

Structurally, MXenes exhibit high specific surface areas, excellent conductivity, hydrophilicity, and mechanical flexibility. Their layers are held together by van der Waals forces, facilitating easy exfoliation and stable dispersion in aqueous media. The abundance of polar surface groups contributes to superior water dispersibility and chemical reactivity, making them ideal platforms for further functionalization with biomolecules [150]. Additionally, MXenes with strong photothermal properties can rapidly elevate local temperatures under NIR irradiation, enabling synergistic photothermal and chemical bactericidal effects, especially useful in microbially dense environments such as the oral cavity and infected wounds [11].

#### 4.4.1. Periodontitis

##### Inhibition of Periodontal Pathogens

Studies have shown that various types of MXene materials can effectively inhibit the growth of multiple pathogenic bacteria under either dark conditions or near-infrared (NIR) irradiation [151]. This broad-spectrum antibacterial activity enables MXenes to be applicable in complex microbial environments, making them particularly suitable for special niches like the oral cavity, which is highly moist and harbors diverse microbial populations.

For MXenes with photothermal properties, such as Ti_3_C_2_T_x_, NIR irradiation enables rapid photothermal conversion, raising the local temperature and accelerating bacterial death—thereby achieving a photothermal–chemical synergistic antibacterial effect. For instance, Yu et al. developed a Ti_3_C_2_T_x_ MXene-based nanosystem loaded with indocyanine green (ICG) (ICG-MXene) for combined photothermal therapy (PTT) and photodynamic therapy (PDT) against MRSA. Through physical adsorption, ICG was efficiently loaded onto the MXene, yielding excellent photothermal heating and ROS-generating capabilities. Under 808 nm NIR irradiation for 5 min, ICG-MXene completely eradicated MRSA with a 100% bactericidal rate, significantly outperforming treatments using ICG or MXene alone [152]. This system exhibits advantages such as low-dose efficiency, high antibacterial potency, and targeting of drug-resistant bacteria, highlighting its promise as an antibiotic-free antibacterial phototherapy platform.

Moreover, the negatively charged groups on the MXene surface (e.g., –OH, –F, –O) facilitate physical adsorption of certain drugs via electrostatic interactions or hydrogen bonding. This non-covalent coupling helps preserve the antibacterial function of the drug while retaining MXenes’ ROS-scavenging and osteoinductive properties [153]. Yu et al. constructed an injectable hydrogel (GPM) incorporating gelatin, Ti_3_C_2_T_x_ MXene, and the cationic antimicrobial peptide polylysine (PL). The hydrogel showed significant antibacterial effects against *Porphyromonas gingivalis*, notably inhibiting colony formation and damaging bacterial morphology—evidenced by cell shrinkage and decreased viability. This effect is mainly due to the electrostatic interactions between PL and the negatively charged bacterial membrane, which promote adhesion and aggregation, followed by membrane insertion, pore formation, structural disruption, and membrane potential collapse, ultimately leading to cytoplasmic leakage, metabolic disturbance, and bacterial death [151].

##### Promotion of Periodontal Bone Regeneration

The key to periodontal tissue regeneration lies in reconstructing functional periodontal attachment structures, especially the formation of new alveolar bone. This process relies on the proliferation, migration, and osteogenic differentiation of periodontal ligament stem cells (PDLSCs) or periodontal fibroblasts. However, under conditions of chronic inflammation, oxidative stress, and persistent bacterial invasion, bone regeneration is often severely impaired. Therefore, the development of novel materials with integrated antibacterial, anti-inflammatory, antioxidative, and osteoinductive properties is critical for achieving effective periodontal bone regeneration.

In recent years, 2D transition metal carbide MXene materials—particularly Ti_3_C_2_T_x_—have demonstrated great potential in oral tissue engineering due to their ultrathin layered structure, high specific surface area, and abundant surface functional groups [154]. Recent studies have confirmed that Ti_3_C_2_T_x_ nanosheets are not only biocompatible but also function as bioactive regulators to promote osteogenic differentiation of human periodontal ligament cells (hPDLCs). Mechanistically, Ti_3_C_2_T_x_ enhances the binding affinity of Wnt-Frizzled receptor complexes, effectively activating the Wnt/β-catenin signaling pathway, stabilizing HIF-1α expression, and driving metabolic reprogramming toward glycolysis. This upregulates key osteogenic transcription factors such as RUNX2, HIF-1α, and β-catenin, thereby synergistically promoting osteogenesis. In a rat periodontal bone defect model, hPDLCs pretreated with Ti_3_C_2_T_x_ significantly improved new bone formation and inhibited osteoclast activity after implantation, confirming the high therapeutic potential of Ti_3_C_2_T_x_ in periodontal tissue regeneration [155,156].

Remarkably, Ti_3_C_2_T_x_ MXene also exhibits superior enzyme-mimetic antioxidant properties due to its hydrophilic functional groups and electron-transfer capabilities. At biocompatible concentrations, it can efficiently scavenge various ROS, including H_2_O_2_, O_2_·^−^, and ·OH. Building on this, Yu et al. developed an injectable nanocomposite hydrogel (GPM) comprising gelatin, Ti_3_C_2_T_x_ MXene, and polylysine (PL), which rapidly gels via enzymatic crosslinking. The hydrogel showed strong antibacterial activity (via PL), effective ROS scavenging (via MXene), and promoted osteogenic gene expression (RUNX2, ALP, OCN) in hPDLCs. In vivo, GPM enhanced bone regeneration and reduced inflammation, offering a promising strategy for minimally invasive periodontitis treatment [151].

**Figure 6:**
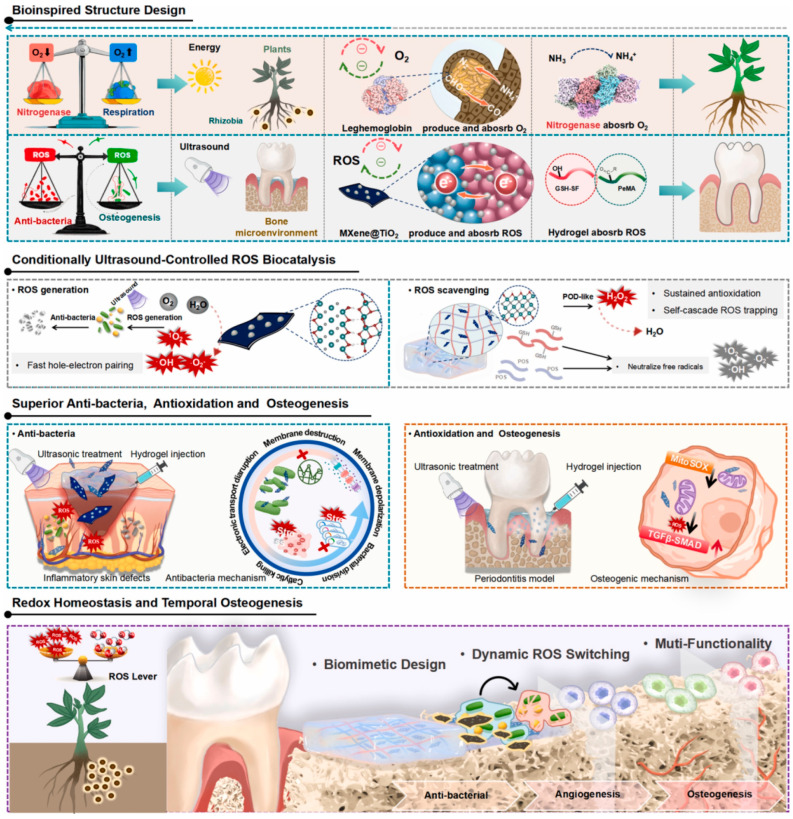
Schematic illustration of bioinspired MXene@TiO_2_ hydrogel design and ROS-regulating strategy for periodontitis treatment, highlighting its multifunctional roles in antibacterial activity, angiogenesis, and osteogenesis [157]. Copyright 2025, Elsevier.

Furthermore, addressing the dual challenges of infection and oxidative stress in periodontal repair, as shown in Figure 6, Xu et al. developed a smart injectable hydrogel (SP-MT hydrogel) based on a MXene@TiO_2_ heterostructure that enables spatiotemporal ROS modulation triggered by ultrasound. Under ultrasound stimulation, the hydrogel efficiently generates •OH and •O_2_^−^, achieving a 98.7% bactericidal rate against *P. gingivalis*. Simultaneously, it exhibits peroxidase-like activity to scavenge excess ROS under unstimulated conditions, restoring redox homeostasis. In vitro, the hydrogel significantly upregulated osteogenic genes (Runx2, OCN), restored mitochondrial function under oxidative damage, and activated the TGF-β/SMAD signaling pathway, promoting osteogenic differentiation of MC3T3-E1 cells. In a periodontitis model, the SP-MT hydrogel group showed higher bone mineral density (BMD), increased bone volume fraction (BV/TV), reduced CEJ-ABC distance, and significantly lower inflammatory cytokine levels (TNF-α, IL-6), providing an innovative platform for precise treatment of chronic infectious bone defects [157].

Additionally, MXene nanomaterials have been successfully integrated into highly ordered ternary nanofiber scaffolds (e.g., PCM scaffolds), whose aligned structures support cell adhesion and spreading. Through upregulation of iNOS and SGK1, these scaffolds regulate intracellular Ca^2+^ levels and activate the mTOR-AKT pathway, promoting differentiation, fusion, and myoskeletal regeneration of C2C12 myoblasts [158]. Similarly, MXene can also serve as a functional nanofiller in 3D bioprinting hydrogels (GHM bioink). When combined with GelMA/HAMA, the resulting hydrogel structure demonstrates excellent printability and porosity. It facilitates upregulation of both early and late myogenic markers without exogenous inducers, promoting the reconstruction and integration of myogenic-to-osteogenic differentiation processes [159].

In summary, MXenes’ advantages in periodontal bone regeneration are reflected in their ability to regulate cellular signaling pathways, reshape the regenerative microenvironment, and simultaneously achieve antibacterial and osteoinductive effects, demonstrating broad application prospects in periodontal regenerative therapy.

#### 4.4.2. Peri-Implantitis

Studies have confirmed that MXene coatings applied on various implant substrates—including titanium, PEEK, zinc, and 316L stainless steel—can significantly suppress the adhesion and growth of *S. aureus*, *E. coli*, and their drug-resistant strains such as MRSA [160]. For example, Ti_3_C_2_T_x_ coatings deposited on CFPEEK substrates, when combined with NIR irradiation, induce both photothermal and ROS-based bactericidal effects (PTT/PDT), while simultaneously promoting adhesion and osteogenic differentiation of bone marrow mesenchymal stem cells (BMSCs), leading to excellent bone regeneration outcomes in cranial defect models [161].

MXenes also demonstrate notable advantages in promoting osteogenesis. Ti_3_C_2_T_x_ coatings upregulate key osteogenic genes such as RUNX2, ALP, and OPN, thereby promoting osteoblastic differentiation. This mechanism may be closely related to the material’s electroactive surface interface, activation of integrin α2β1-mediated signaling, and modulation of the MEK/ERK and Wnt/β-catenin pathways. Furthermore, MXene coatings possess suitable surface roughness and hydrophilicity, which facilitate BMSC adhesion [162]. Guan et al. developed a multifunctional coating (Gel@MX-ZIF8/CA) composed of MXene, ZIF-8, and GelMA. Through synergistic mechanisms, this coating significantly enhances bone regeneration. MXenes, by virtue of their 2D structure and surface functionalities, activates the Wnt/β-catenin signaling pathway and glycolytic metabolism, promoting osteoblast differentiation and mineralization. ZIF-8 undergoes acid-responsive degradation, releasing Zn^2+^ ions that activate VEGF and BMP signaling, thus enhancing angiogenesis, regulating the immune microenvironment, suppressing inflammation, and improving bone healing. The GelMA hydrogel serves as a sustained drug release platform to ensure the effective delivery of cinnamaldehyde (CA) and Zn^2+^. Together, these mechanisms accelerate bone formation and improve implant integration and biocompatibility, offering an innovative strategy for bone repair [163].

Currently, guided bone regeneration (GBR) is a widely used technique in implant therapy. By creating a closed space that prevents the ingrowth of rapidly proliferating fibrous connective tissue into bone defect areas, GBR promotes bone regeneration. Zhang et al. evaluated the osteogenic effect and mechanism of Ti_3_C_2_T_x_ MXene films in GBR. The results showed that MXene films significantly promoted the adhesion, proliferation, and osteogenic differentiation of pre-osteoblasts. Especially in early stages, they enhanced alkaline phosphatase (ALP) activity and upregulated osteogenic genes such as ALP, OCN, and OPN, thereby facilitating osteogenesis. Due to their excellent biocompatibility and osteoinductive properties, MXene films present as promising materials for bone tissue engineering and guided bone regeneration therapies [164].

MXenes also possess favorable immunomodulatory properties. In infected environments, MXene coatings can induce macrophage polarization toward the M2 phenotype, regulate the expression of inflammatory cytokines such as TNF-α and IL-6, and upregulate IL-10, thereby suppressing inflammation, enhancing tissue homeostasis, and accelerating repair [165]. Asadi et al. reported that a MXene/HAP composite with a micro-wrinkled structure not only promotes osteoblast function but also improves immune compatibility. The micro-scale wrinkles effectively guide macrophages toward an anti-inflammatory, pro-healing M2 phenotype, reduce inflammation, and enhance tissue repair. This immunomodulatory effect is achieved by activating the RhoA/ROCK signaling pathway, thus creating a favorable immune environment for implant integration and tissue regeneration [166].

In terms of physical properties, the incorporation of MXenes significantly enhances the wear resistance of composite materials. Rothammer et al. found that adding Ti_3_C_2_T_x_ into a UHMWPE matrix reduced the friction coefficient and wear rate by 19% and 44%, respectively, providing better mechanical stability for load-bearing implants [167]. Furthermore, Ma et al. developed a novel PCL-MXene coating for magnesium implants to control their degradation. Although magnesium implants are biocompatible, their rapid degradation can lead to implant fracture. The coating protects the implant during the early healing phase and enables controlled degradation under NIR stimulation once the bone has regained sufficient strength. Leveraging MXenes’ photothermal properties, the coating rapidly heats and degrades under NIR light, exposing the magnesium substrate. This approach offers a new solution for the controlled degradation of magnesium-based implants [163].

In conclusion, MXenes, as a new generation of implant coating material, integrate hydrophilicity, antibacterial activity, photothermal responsiveness, osteoinductive capacity, immunomodulatory functions, controlled release capability, and excellent mechanical performance. They offer a comprehensive strategy to address the challenges of implant-associated infection and osseointegration. Future efforts should focus on green synthesis, intelligent structural design, and validation in preclinical models to accelerate clinical translation in the field of dental implants.

### 4.5. Transition Metal Dichalcogenides (TMDs)

Transition Metal Dichalcogenides (TMDs) are two-dimensional (2D) layered nanomaterials composed of a transition metal (such a12s molybdenum or tungsten) and chalcogen elements (sulfur, selenium, or tellurium) [126]. Owing to their unique optoelectronic properties, tunable bandgap, high surface area, and favorable biocompatibility, TMDs have gained increasing attention in biomedical research. One of their most notable features is efficient photothermal conversion: under near-infrared (NIR) irradiation, TMDs absorb and convert light into heat, enabling precise, minimally invasive photothermal therapy (PTT) with superior efficiency and compatibility compared to graphene [168].

Beyond PTT, TMDs can synergize with photodynamic and immunotherapies by inducing apoptosis and activating immune responses, while their abundant functional groups facilitate high drug loading and controlled, pH-responsive release for targeted therapy [169]. Molybdenum disulfide (MoS_2_) quantum dots, in particular, have been studied as nanocarriers for chemotherapeutics and photosensitizers, where surface modifications enhance stability, circulation, and targeting. Furthermore, incorporation of MoS_2_ nanosheets into bioceramic scaffolds improves mechanical stability and promotes osteogenic differentiation, providing promising strategies for bone regeneration in tumor-related defects [170].

#### Periodontitis

**Figure 7:**
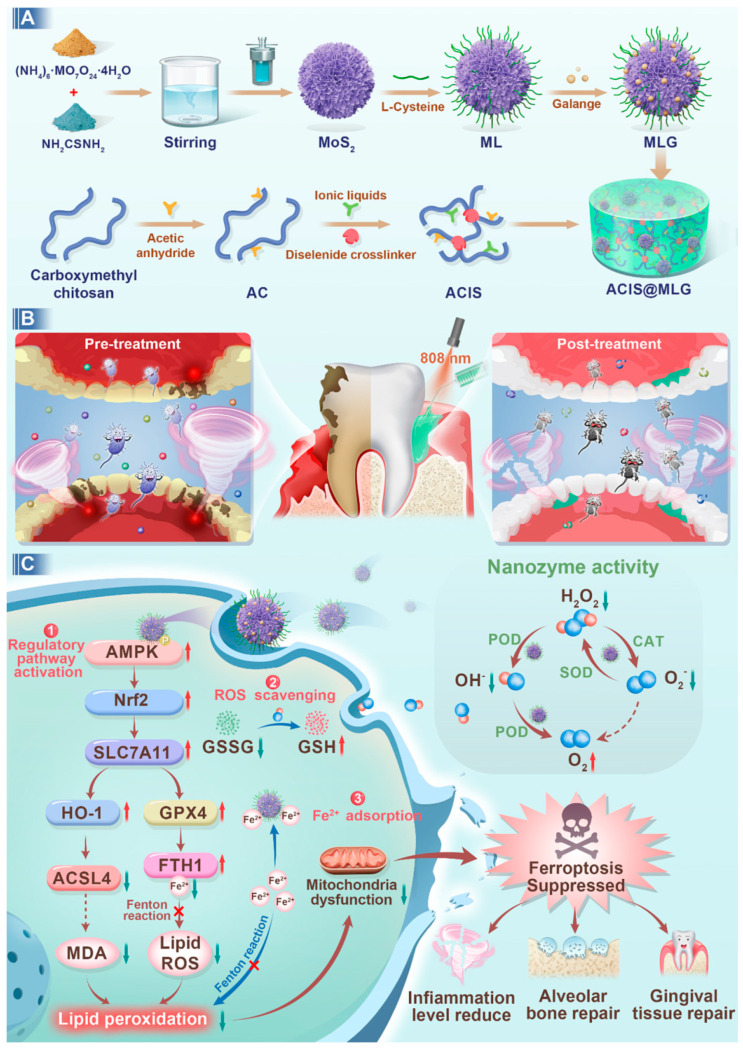
Schematic illustration of the multifunctional ACIS@MLG platform for periodontitis treatment. (**A**) Synthetic route of ACIS@MLG. (**B**) After administration into the periodontal pocket, ACIS@MLG forms a thermosensitive hydrogel that delivers antibacterial, anti-inflammatory, and protective effects to periodontal tissues. (**C**) Proposed mechanism of ferroptosis inhibition: MLG adsorbs free Fe^2+^ and scavenges ROS to block ferroptosis, while modulating lipid peroxidation through the AMPK/Nrf2/SLC7A11 signaling pathway [171]. Copyright 2025, Elsevier.

Although research on TMDs in periodontitis remains limited, recent studies have revealed promising multifunctional activities. As shown in Figure 7, Tang et al. developed a MoS_2_ nanoflower modified with L-cysteine and galangin (MLG), embedded into a thermosensitive ionic liquid hydrogel (ACIS), forming a multifunctional nanozyme hydrogel (ACIS@MLG) designed to modulate ferroptosis for treating periodontitis. In rat models with periodontitis induced by pathogens and LPS, the hydrogel exhibited significant antibacterial, anti-inflammatory, and tissue-protective effects. Mechanistically, MLG scavenged ROS and reduced intracellular iron load, restoring mitochondrial membrane potential disrupted by ferroptosis. It also activated the AMPK/Nrf2/SLC7A11 pathway to regulate lipid peroxidation, suppressing ferroptosis in periodontal ligament stem cells and promoting tissue homeostasis. This study introduced a ferroptosis-based strategy for periodontitis therapy using MoS_2_ nanomaterials [171].

Beyond periodontitis, TMDs have shown great potential in treating other inflammatory diseases, particularly osteoarthritis [172]. For example, Zhao et al. created a chitosan-functionalized MoS_2_ nanosheet system loaded with dexamethasone (MCD), which under NIR irradiation exhibited significant photothermal-triggered anti-inflammatory effects. MoS_2_ enabled localized heating, controlled drug release, and improved cellular uptake and endosomal escape, enhancing intracellular drug bioavailability. The MCD+NIR system promoted macrophage apoptosis and suppressed key proinflammatory cytokines such as TNF-α, IL-1β, and IL-8 [173].

In addition to photothermal drug release, MoS_2_ has demonstrated antioxidant capabilities. Chen et al. reported fullerene-like MoS_2_ nanoparticles (F-MoS_2_) that mimic enzyme activities, possessing both SOD-like and CAT-like functions. These particles form a cascade catalytic system capable of scavenging superoxide and hydrogen peroxide. By leveraging Mo^6+^/Mo^4+^ redox cycling, F-MoS_2_ efficiently removes ROS, protects hyaluronic acid from oxidative degradation, and improves cell survival under oxidative stress [174]. These multifunctional properties suggest strong potential for TMDs in ROS-related diseases, including inflammatory oral conditions like periodontitis.

### 4.6. Layered Double Hydroxides (LDHs)

Layered double hydroxides (LDHs) are two-dimensional inorganic nanomaterials composed of divalent and trivalent metal cations together with interlayer anions, featuring tunable layered structures, high surface area, and favorable biocompatibility. Their unique configuration makes them ideal carriers for small-molecule drugs, siRNA, and natural bioactives, with controlled release and responsiveness to pathological microenvironments [175]. Under acidic, H_2_O_2_-rich, or ROS-elevated conditions, LDHs enable site-specific delivery while the released metal ions exert intrinsic antibacterial, anti-inflammatory, osteogenic, and antioxidant activities, providing opportunities for drug-free therapeutic strategies [176]. Moreover, their surface can be readily modified and integrated with hydrogels or biomacromolecules, enhancing functional adaptability in complex oral microenvironments.

#### 4.6.1. Periodontitis

Bacterial biofilm formation is a primary initiator of periodontitis. Copper–aluminum LDH (CuAl-LDH) is a functional variant known for its intrinsic Fenton-like catalytic activity, which enables efficient generation of hydroxyl radicals (·OH) in the presence of H_2_O_2_. This oxidative stress severely damages bacterial membranes, significantly reduces the viability of key oral pathogens such as *P. gingivalis* and *F. nucleatum*, and disrupts established multispecies biofilms, thereby reducing pathogenic burden [15]. In addition, divalent metal ions such as Zn^2+^ and Mg^2+^ have been recognized for their ability to stimulate osteoblast activity, upregulate osteogenic gene expression, and enhance mineralization [177]. LDHs releasing such ions have been shown to promote the proliferation and differentiation of osteogenic cells including bone marrow-derived mesenchymal stem cells (BMSCs) and human periodontal ligament stem cells (hPDLSCs), while simultaneously suppressing osteoclastogenesis and the secretion of pro-inflammatory cytokines, achieving dual anti-inflammatory and bone regenerative effects [178].

A recent study developed a multifunctional composite system by incorporating icariin (ICA)-loaded ZnAl-LDH nanosheets into a modified chitosan hydrogel (GA-HBC), termed GA-HBC-LIC. This platform enabled a temporally and spatially coordinated treatment of periodontitis. In the early phase, the synergistic antibacterial activity of chitosan, gallic acid, and Zn^2+^ effectively reduced the oral pathogenic load. Subsequently, Zn^2+^ triggered the activation of the transcription factor ZEB1, driving macrophage polarization from the pro-inflammatory M1 to the pro-regenerative M2 phenotype, thereby remodeling the immune microenvironment in favor of tissue healing. Following inflammation resolution and oxidative stress attenuation, ICA was gradually released from the LDH carrier, continuously activating osteogenic transcription factors such as RUNX2 and SP7, promoting the proliferation and differentiation of osteoprogenitors, and enhancing the regeneration of bone and periodontal supporting structures [179]. Moreover, LDHs intercalated with NO_3_^−^ have been reported to modulate immune responses via induction of CX3CR1^+^ anti-inflammatory macrophages and inhibition of Th17 cell activation and IL-17 signaling, thereby achieving immune regulation without pharmaceutical agents [180].

The potential of LDHs as long-term drug delivery systems has also been validated. For example, MgAl-LDHs have been employed to load non-steroidal anti-inflammatory drugs (NSAIDs), including naproxen and diclofenac. By adjusting the crystalline structure and particle size, a controlled release profile was achieved, enabling prolonged drug activity at the lesion site and offering a promising strategy for chronic periodontitis management [181]. In another study, LDHs were integrated into a PNIPAM-based thermoresponsive hydrogel for siRNA delivery. This system facilitated gene silencing of inflammatory targets in chondrocytes and exhibited strong anti-inflammatory effects and biocompatibility in vivo, highlighting its potential for precise intervention in inflammatory disease progression [182].

#### 4.6.2. Dental Implants

In addition to periodontitis therapy, LDHs have also demonstrated great potential in the surface modification of dental implants and bone tissue engineering. As two-dimensional materials with tunable structures and controllable ion release, LDHs not only exhibit excellent biocompatibility and sustained release behavior but can also construct biofunctional interfaces for multipathway regulation of the bone microenvironment, making them highly valuable in implant-based rehabilitation [178].

**Figure 8:**
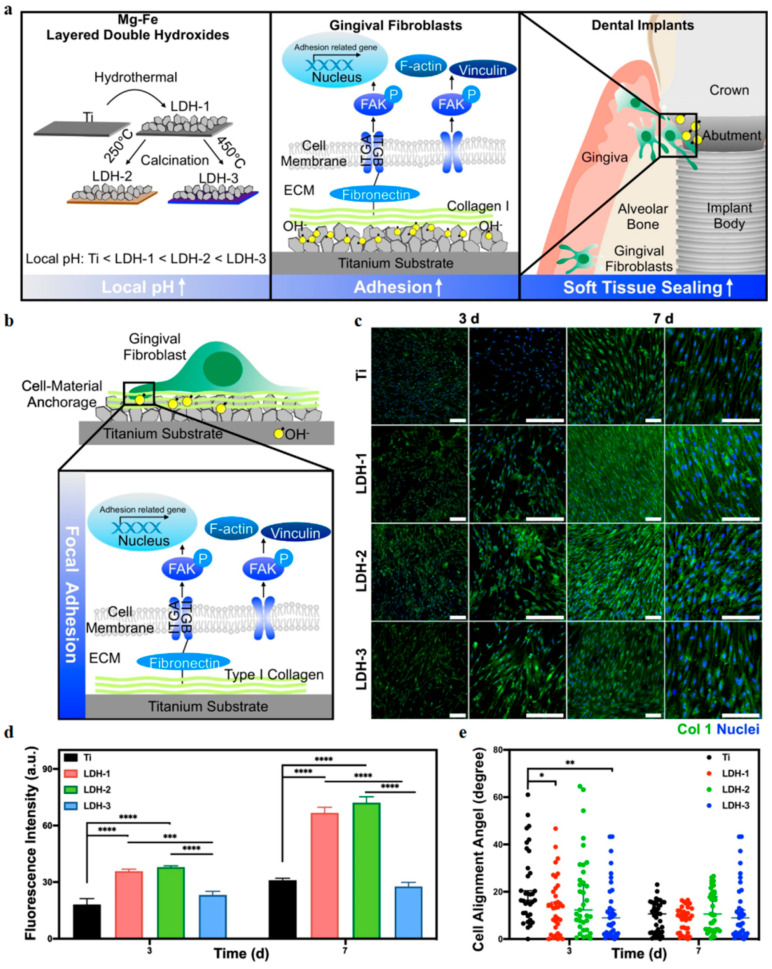
Enhanced adhesion and collagen expression of gingival fibroblasts on Mg–Fe LDH films. (**a**) Schematic illustration of how Mg–Fe LDH films enhance gingival fibroblast adhesion by regulating local pH; (**b**) Diagram showing ECM–integrin–cytoskeleton link formation under alkaline conditions; (**c**) Confocal images of hGFs on different samples at 3 and 7 days, stained for type I collagen (green) and nuclei (blue, DAPI); (**d**) Quantification of type I collagen fluorescence intensity at 3 and 7 days; (**e**) Cell alignment angle distribution on each surface at 3 and 7 days. Statistical differences: * *p* < 0.05, ** *p* < 0.01, *** *p* < 0.001, **** *p* < 0.0001 [184]. Copyright 2021, Elsevier.

Recent studies have shown that LDHs can function as osteoconductive coatings on titanium surfaces. A MgFe-LDH coating, prepared via in situ growth, was found to create a stable mildly alkaline interface (pH > 8) that significantly suppressed osteoclast activity while promoting osteoblast differentiation and extracellular matrix deposition, thereby enhancing osseointegration [183]. Additionally, as shown in Figure 8, Yin et al. found that this coating promoted the adhesion, cytoskeletal reorganization, and extracellular matrix secretion of human gingival fibroblasts. By upregulating adhesion-related genes such as COL1A1, fibronectin (FN), ITGA2, and ITGB1 and activating the focal adhesion kinase (FAK) signaling pathway, it reinforced the stability of the cell–material interface, contributing to the formation of a soft tissue seal and reducing the risk of peri-implantitis [184].

Further studies have demonstrated that LDHs can also participate in regulating inflammatory responses and tissue regeneration at the implant interface through immunomodulatory mechanisms. Liang et al. developed an LPS-functionalized MgFe-LDH coating that exhibited a sequential activation effect: it initially promoted M1 macrophage polarization to rapidly eliminate bacterial contamination during the early implantation stage, and subsequently induced M2 macrophage polarization to enhance anti-inflammatory cytokine release and tissue repair. This strategy helped establish a stable immune microenvironment, significantly improving long-term implant stability and osseointegration quality [185]. In bone tissue engineering, LDHs can promote dual regulation of osteogenesis and anti-inflammation due to their controlled release of metal ions such as Mg^2+^, Zn^2+^, Fe^3+^, and Sr^2+^. These ions activate multiple osteogenic signaling pathways, including Wnt/β-catenin and PI3K/Akt, thereby promoting the differentiation of stem cells like BMSCs into osteoblast-like cells. At the same time, LDHs help reduce excess reactive oxygen species (ROS) and inflammatory cytokines and inhibit osteoclast activity [186].

In addition, for biodegradable metallic materials such as magnesium alloys, LDHs have shown potential as surface coatings to improve corrosion resistance [187]. Cheng et al. demonstrated that constructing a MgAl-LDH coating significantly reduced the electrochemical corrosion of magnesium alloys in body fluid environments, improved material stability, and enhanced both new bone formation and angiogenesis. This has important implications for expanding the clinical applications of Mg-based implants [188].

## 5. Advantages and Improvement

With the widespread application of two-dimensional (2D) nanomaterials in the medical field, significant progress has been made in their research for the treatment of oral diseases. Previous studies have systematically summarized their multiple mechanisms in diseases such as dental caries, periodontitis, oral cancer, and peri-implantitis, including antibacterial, anti-inflammatory, ROS scavenging, and tissue regeneration properties. As a new material system with high potential for functional integration, 2D nanomaterials demonstrate therapeutic performance and design space far beyond traditional carrier platforms. However, to achieve their widespread clinical application, it is necessary to systematically address the real challenges they face in terms of biological safety, stability, targeting efficiency, and large-scale translation.

### 5.1. Multifunctional Integration Advantage for Adapting to Complex Oral Pathology

The most prominent feature of 2D materials is their ability to integrate multiple functions synergistically. Oral diseases are often characterized by complex etiology, multi-stage disease progression, and short treatment intervention windows, making it difficult to achieve comprehensive control through a single mechanism. In contrast, 2D materials can simultaneously achieve multiple functions such as antibacterial, anti-inflammatory, antioxidant, and promotion of hard and soft tissue regeneration on a single nanoplatform. For example, GO can destroy bacterial membrane structures through its sharp nanosheets, adsorb antibacterial drugs, and modulate immune responses [189]. MXene materials combine photothermal sterilization and ROS scavenging effects, with excellent induction capabilities in bone tissue regeneration [151]. Black phosphorus nanosheets degrade under light to generate phosphate ions, maintaining a mineralized microenvironment while slowly releasing them [190]. The high specific surface area of these materials not only enhances their adhesion to tissue interfaces but also significantly increases the loading density and local effect intensity of drug molecules or signaling factors. By integrating smart responsive strategies (e.g., pH, enzyme, ROS response) and surface modifications (e.g., HA, RGD peptides, targeting ligands), disease-specific recognition and controlled release can be achieved, providing “spatial-temporal precise intervention” in the early stages of caries, chronic inflammation, or tumor tissues [191,192,193,194]. This highly modular therapeutic platform not only improves treatment efficiency but also significantly reduces systemic side effects, providing a theoretical foundation and material support for future personalized and multi-target combination therapies.

### 5.2. Biological Safety Still Need Comprehensive Evaluation

Although 2D materials have demonstrated good biocompatibility in short-term animal experiments, their long-term safety in the complex oral environment remains controversial. Due to their highly active surfaces and nanoscale characteristics, these materials are prone to form a “protein corona” by binding with proteins in body fluids, which affects their biodistribution, cellular uptake, and immune recognition pathways, potentially triggering chronic inflammation or nonspecific toxicity [195,196]. Importantly, the potential for persistent inflammatory responses caused by non-degradable or slowly degrading 2D nanomaterials in the oral mucosa has not been sufficiently addressed. Repeated or long-term exposure could lead to immune cell infiltration, fibrosis, and delayed wound healing, representing a significant barrier to clinical translation. In addition, some inorganic 2D materials, such as MXene and TMDs, may release metal ions or charged intermediate products during degradation, inducing oxidative stress or cytotoxic reactions, especially in sensitive sites such as the gingival mucosa. Although BP is biodegradable, its rapid hydrolysis may result in a short-lived, unstable therapeutic effect. In addition, some materials still require the use of highly toxic reagents (such as HF and strong oxidants) during synthesis, and if purification is inadequate or impurities remain, they may cause toxic irritation to cells. To improve clinical acceptability, future research should focus on green synthesis strategies, the development of biodegradable coating systems, and systemic studies on long-term toxicity, immune responses, and metabolic transport pathways, particularly establishing standardized evaluation models for practical scenarios such as long-term contact with oral mucosa, swallowing, and trace blood entry [197].

### 5.3. Structural Stability and Functional Maintenance Are Still Limited

The physicochemical stability of 2D materials is a key indicator of whether they can maintain functional continuity in practical applications. Many materials, due to their high surface energy and active edge structures, are prone to oxidation, agglomeration, or sheet breakage during storage and application, resulting in a loss of activity [198]. These phenomena may lead to uncontrolled drug release, reduced biocompatibility, or even inactivation. The high humidity, salivary enzyme activity, and frequent mechanical stress in the oral environment further exacerbate the challenges to material structure. To address stability issues, some studies have attempted to enhance material stability and environmental tolerance through surface coatings (such as PEG, PVP, gelatin), ionic crosslinking, covalent encapsulation, or core–shell structures. In addition, structural regulation (such as optimizing layer numbers, doping, and heterojunction design) can alleviate surface reactivity and extend the effective service life of materials [199]. Future material design should combine “physicochemical stability + biological functionality” requirements to further enhance their sustainability in clinical oral environments.

### 5.4. Clinical Translation Challenges Remain to Be Overcome

Currently, the delivery efficiency and targeting precision of 2D materials still need improvement. Although various responsive or ligand modification strategies have been developed to enhance their accumulation in subgingival inflammation, tumor microenvironments, or bacterial membranes, their permeability, adhesion, and release accuracy in complex three-dimensional structures, such as peri-implant gingival sulci in multi-bacterial co-infection, still fail to meet the demands of efficient treatment [200]. Additionally, the preparation and application of 2D materials still face large-scale challenges. Most materials currently rely on laboratory-based liquid-phase exfoliation, CVD, intercalation reactions, etc., making it difficult to achieve standardized, low-cost, and large-scale production. Issues such as large inter-batch differences, uneven layer control, and unstable functional group modifications severely limit their feasibility as registerable medical materials. Meanwhile, the regulatory approval pathway for 2D materials remains unclear, with a lack of well-defined toxicological standards, functional evaluation metrics, and industrial translation routes. At present, a unified regulatory framework is still lacking; therefore, it is necessary to propose potential classification approaches, such as defining these materials according to their primary mode of action. One potential approach is to classify these materials according to their primary mode of action: for example, implant coatings, barrier membranes, or scaffolds would likely be considered implantable medical devices; nanomaterials directly administered into the body could be regulated as drugs or drug delivery systems; and multifunctional platforms that combine diagnostic and therapeutic roles may fall into the category of combination products. When incorporated into restorative materials such as composites or cements, they may be regulated as dental restorative devices. Particularly in the oral field, materials need to form integrable and replaceable product forms with existing dental instruments, restorative systems, or drug delivery systems to truly enter the clinical application process. Particularly in the oral field, materials need to form integrable and replaceable product forms with existing dental instruments, restorative systems, or drug delivery systems to truly enter the clinical application process. Therefore, establishing a transformation platform that integrates “preparation–evaluation–application–regulation” is key to transitioning 2D materials from the laboratory to the clinic. The typical advantages and disadvantages of 2D nanomaterials are summarized in Table 2.

## 6. Conclusions

Two-dimensional (2D) nanomaterials have gradually emerged as cutting-edge platforms for precise intervention in oral diseases, owing to their unique physicochemical properties. Compared to traditional 0D, 1D, or 3D nanostructures, 2D materials—featuring ultrathin layered structures, tunable specific surface areas, and abundant surface functional groups—exhibit superior functional integration and adaptability across multiple therapeutic domains, including antibacterial activity, drug delivery, and tissue regeneration. Particularly in the oral cavity, a highly dynamic and microbially complex ecosystem prone to contamination, the interfacial reactivity and synergistic therapeutic potential of 2D materials offer promising translational prospects.

Compared with 3D nanostructures, 2D materials provide larger planar contact interfaces, facilitating tight interactions with bacterial membranes, cellular membranes, and tissue interfaces. This enhances both antibacterial penetration and drug delivery efficiency. Representative materials such as GO, MXene, and BP demonstrate excellent photothermal conversion and ROS-regulating capabilities, enabling noninvasive, targeted therapy under near-infrared (NIR) light, thereby overcoming the limitations of antibiotic resistance and systemic toxicity associated with conventional treatments. Additionally, materials like 2D MOFs and LDHs possess ion-releasing and pH-modulating properties, exhibiting strong bioactivity in bone and soft tissue regeneration, and offering multi-modal support for complex oral tissue repair.

This review systematically summarizes current research on the application of 2D nanomaterials in various representative oral diseases, including dental caries, periodontitis, oral cancer, and peri-implant infections. It focuses on their multifunctional therapeutic mechanisms and structure–activity relationships in bacterial eradication, biofilm disruption, immune microenvironment modulation, oxidative stress relief, and bone regeneration. These studies lay a solid mechanistic foundation for the oral application of 2D nanomaterials. However, it is important to note that most of these advances remain in the laboratory stage, and significant challenges persist before clinical translation can be realized. Comprehensive evaluations of long-term biocompatibility, degradation and metabolic pathways, chronic toxicity, and immunogenicity are still lacking—especially regarding the safety and therapeutic durability of 2D materials in the complex oral environment.

Future research should focus on advancing 2D materials from functional proof-of-concept to clinically translatable product development. First, materials with enhanced clinical adaptability should be designed, featuring degradability, targeting capability, and environmental responsiveness to enable precise intervention at specific lesion sites. Second, efforts should be made to green, modularize, and scale up synthesis processes to ensure manufacturability and batch consistency, thereby laying a technological foundation for clinical registration and industrial translation. Moreover, exploring the combination of 2D materials with traditional treatment approaches—such as periodontal surgery, light-curable resins, or chemo-radiotherapy—may further expand their clinical utility.

In summary, 2D nanomaterials are leading a paradigm shift in oral disease treatment—from passive intervention to intelligent, multi-mechanism precision therapy. With the continued integration of materials science, oral medicine, and bioengineering, future 2D material-based personalized therapeutic platforms are expected to achieve breakthroughs in early disease intervention, recurrence prevention, and tissue regeneration, reshaping both the scientific and practical landscape of precision oral healthcare.

## Figures and Tables

**Table 1:** Applications of Graphene and Its Derivatives in Oral Diseases.

Diseases	Materials	Author	Methods	Mechanism
Dental Caries	Ag@nGO	Arslan et al. [75]	Silver nanoparticles synthesized by chamomile extract (biogenic) or chemical methods were immobilized on nano-graphene oxide (nGO) to form Ag@nGO NCs, which were then added (0.05% w/w) into dental adhesives (Clearfil SE Bond).	Ag^+^ release combined with graphene-induced membrane damage and ROS generation provided strong antibacterial and antibiofilm effects against oral pathogens.
	GO-Cu NCs	Mao et al. [76]	Copper nanoparticles are anchored onto graphene oxide to form stable nanocomposites with sustained Cu^2+^ release.	Disrupting S. mutans biofilm formation by impairing EPS matrix synthesis, downregulating gtfB/C, gbpB, upregulating rnc, and interfering with carbohydrate metabolism, while maintaining good biocompatibility.
	AGONSs	Lu et al. [77]	Ethylenediamine-modified GO nanosheets with enhanced photothermal activity	Kills S. mutans via positive charge binding, membrane cutting, and NIR-induced photothermal/ROS effects.
	BAG@GO	Lee et al. [78]	BAG synthesized by sol–gel method and combined with GO, then incorporated at 1%, 3%, 5% into orthodontic adhesives. Mechanical, antibacterial, and anti-demineralization properties were tested.	BAG released Ca^2+^/PO_4_^3−^ ions to buffer acidity and promote remineralization, while GO provided antibacterial effects via oxidative stress and bacterial membrane damage. Together, BAG@GO adhesives showed improved microhardness, anti-demineralization, and antibacterial efficacy.
	MBN@GOQD	Son et al. [79]	MBN synthesized by modified sol–gel method and subsequently coated with GOQD using colloidal processing.	GOQD promotes rapid nucleation and deposition of hydroxyapatite while maintaining Ca, Si, and P ion release, thereby enhancing remineralization and effectively sealing dentinal tubules for desensitization.
	FG	Shaheen et al. [80]	FG nanosheets were synthesized by hydrothermal method and prepared as a gel	FG significantly increased the microhardness of enamel (especially at depths of 100–150 μm) and improved the Ca/P ratio, as well as the color (ΔE00); its mechanism is related to fluoride doping promoting the formation of fluorapatite, enhancing acid resistance and penetration. n-HAp primarily promotes surface remineralization by providing Ca^2+^ and PO_4_^3−^ ions.
Oral Squamous Cell Carcinoma (OSCC)	GO-PEI-miR-214	Ou et al. [89]	Functionalized GO with PEI used for miRNA inhibitor delivery	The complex prevents the progression of OSCC by suppressing miR-214 levels, leading to increased expression of PTEN and p53, and inhibiting the PI3K/Akt pathway. It shows strong anticancer effects both in vitro and in vivo without affecting organ tissues.
	PEG-GQDs-Pt (GPt)	Wei et al. [90]	Graphene quantum dots synthesized by chemical oxidation, covalently bound with cisplatin (Pt) and PEGylated	Overcomes hypoxia-induced chemoresistance by enhancing Pt accumulation, inducing S-phase arrest and apoptosis, and increasing tumor targeting via EPR effect, thereby inhibiting OSCC growth with reduced systemic toxicity.
	DOX@NGO-BBN-AF750	Li et al. [91]	Carboxylated nano-graphene oxide (NGO) non-covalently coupled with bombesin antagonist peptide (BBN-AF750) and doxorubicin (DOX) through π–π stacking and hydrogen bonding	GRPR-targeted and pH-responsive nanocarrier enabling imaging-guided therapy; promotes tumor-specific DOX release, enhances uptake by HSC-3 cells, shows dose- and pH-dependent cytotoxicity, improves stability, prolongs drug half-life, and achieves effective OSCC inhibition.
	AGO	Chen et al. [92]	Graphene oxide (GO) modified with amino groups through chemical reaction with ethylenediamine	AGO demonstrates significantly enhanced photothermal effects under near-infrared (NIR) irradiation, with improved cell uptake and retention in tumor tissues. It induces cell apoptosis and effectively inhibits tumor growth both in vitro (HSC-3 cells) and in vivo (tumor-bearing mice), without affecting normal tissues. AGO is promising for photothermal therapy (PTT) of oral squamous cell carcinoma (OSCC).
	DOX@GO-HA-HN-1	Li et al. [93]	Graphene oxide (GO) functionalized with hyaluronic acid (HA) and HN-1 peptide for dual-targeted drug delivery	The system synergistically enhances drug delivery via HA/CD44 and HN-1 targeting, while NIR irradiation promotes localized DOX release and photothermal therapy (PTT), improving OSCC treatment efficacy. In vitro and in vivo results demonstrate high targeting efficiency, reduced toxicity, and enhanced therapeutic outcomes.
	Graphene oxide(GO)+SB-204990+DOX	Li et al. [94]	SB-204990 and DOX loaded onto carboxylated graphene oxide nanoplatform (NSD) through hydrogen bonding and π-π stacking	Triple therapy (lipid starvation, chemotherapy, and photothermal therapy) synergistically increases intracellular drug concentration, significantly inhibiting tumor growth. Photothermal therapy enhances drug effectiveness and reduces chemotherapy resistance.
	GQD-PEG	Zhang et al. [98]	Graphene Quantum Dots (GQDs) covalently bonded with Polyethylene Glycol (PEG) through chemical reactions	The composite material generates singlet oxygen (^1^O_2_) under light activation, inducing strong phototoxicity and leading to OSCC cell apoptosis. In vivo, it enhances tumor accumulation via the EPR effect, provides targeted therapy, reduces systemic toxicity, and triggers significant host immune responses with increased CD8 T cells and pro-inflammatory cytokines (such as IFN-γ, TNF-α), showing excellent anti-tumor effects.
	NGOD-AA	Shi et al. [99]	NGODs synthesized from natural graphite via modified Hummers’ method, NH_3_ annealing, nitric acid oxidation, and hydrothermal treatment in NH_4_OH; combined with AA as a hole scavenger	NGODs (∼4.4 nm, highly crystalline) act as photosensitizers for PDT; AA scavenges photogenerated holes, shifting mechanism from Type-II (^1^O_2_) to Type-I, efficiently producing H_2_O_2_ under white light. This selectively killed oral cancer (OECM-1), lung (PC-9), head & neck (HONE-1), and colon (HCT-116) cancer cells via apoptosis and necrosis, while sparing normal fibroblasts and keratinocytes, showing high biocompatibility and tumor selectivity.
Periodontitis	DNA-aptamer-NGO	Pourhajibagher et al. [105]	NGO synthesized via modified Hummer’s method, then conjugated with FAM-labeled DNA aptamer specific to *P. gingivalis*	Targeted aPDT under 980 nm diode laser irradiation induced ROS generation, reduced *P. gingivalis* viability by 4.33 Log_10_, disrupted biofilms, suppressed virulence gene expression (fimA, rgpA), upregulated oxidative stress gene (oxyR), and decreased metabolic activity, while showing low cytotoxicity and hemocompatibility.
	PCL-GO	Park et al. [107]	3D printed PCL scaffold fabricated by melt-extrusion, surface treated with oxygen plasma for 5 min, then dip-coated with GO solution (0.125–0.5 mg/mL) prepared via modified Hummer’s method	GO coating with plasma treatment enhanced scaffold hydrophilicity, protein adsorption, and PDLSC adhesion, significantly promoted osteogenic differentiation (increased ALP activity, calcium deposition, and osteopontin expression), improved osteoconductivity while reducing GO consumption compared to polymer blending.
	GO Scaffold	Kawamoto et al. [108]	Graphene oxide (GO) was dispersed onto a 3D collagen scaffold using a GO dispersion method	The GO scaffold enhances periodontal tissue healing in dog class II furcation defects. It promotes bone regeneration, formation of periodontal ligament-like and cementum-like tissues, and enhances cell migration and proliferation. The GO scaffold shows improved tissue formation compared to untreated scaffolds with minimal cytotoxicity.
	PGO-PHA-AG Scaffold	Li et al. [109]	Reduced graphene oxide (GO) and hydroxyapatite (PHA) were co-functionalized with polydopamine (PDA) to form a conductive scaffold.	The scaffold possesses multiple functions including antioxidative properties, immunomodulation, and conductivity. It regulates the diabetic periodontal microenvironment, promotes osteogenic differentiation of BMSCs, reduces M1 macrophage polarization, activates M2 macrophages to secrete osteogenesis-related cytokines, and consequently promotes periodontal bone regeneration.
Peri-implantitis	TiGD/TiGV	Agarwalla et al. [110]	Graphene coating was deposited on titanium surfaces using chemical vapor deposition with a vacuum-assisted technique. The coating process was repeated 2 times (TiGD) or 5 times (TiGV).	The graphene coating significantly inhibited Candida albicans biofilm formation and hyphal growth. The study found that, regardless of the number of layers, the graphene nanocoating effectively prevented mature biofilm formation, reduced microbial attachment, and hindered biofilm maturation. This coating strategy offers long-lasting effects in preventing microbial attachment without relying on antibiotics.
	Ti-0.125G	Wei et al. [111]	Graphene powder was mixed with titanium powder, followed by ultrasonic dispersion and ball milling. The mixture was sintered under vacuum at 900 °C and 50 MPa, resulting in a graphene-reinforced titanium composite.	Combines graphene and titanium to enhance antibacterial properties and soft tissue integration for dental implants. It significantly reduces bacterial biofilm formation (e.g., *S. mutans, F. nucleatum, P. gingivalis*), promotes gingival fibroblast (HGF) adhesion, proliferation, and migration, and improves soft tissue sealing without compromising bioactivity. The mechanism involves electron transfer disrupting bacterial respiration and decreasing microbial vitality.
	Graphene Oxide-Minocycline Composite Coating	Liu et al. [112]	Electrochemical deposition and liquid-phase deposition techniques	The coating is synthesized by depositing graphene oxide (GO) on the surface of ultrafine-grained titanium and loading minocycline (MC) on it. The coating demonstrates antibacterial, osteogenic, and anti-inflammatory effects. The experiments show that the coating exhibits significant antibacterial properties against Staphylococcus aureus and effectively inhibits microbial adhesion and biofilm formation, with no significant toxicity to osteoblasts. It enhances the long-term stability and antibacterial capacity of the implant.
	Graphene-Coated Titanium Sheets	Lu et al. [116]	The titanium sheets, treated with SLA, were coated with graphene (rGO) through chemical reduction and modified chemically to enhance their ability to adsorb growth factors	The graphene coating enhanced the osteogenic capability of the titanium sheets by adsorbing and sustainedly releasing concentrated growth factors, promoting osteogenic differentiation of bone marrow stromal cells (BMSCs), and activating the RhoA/ROCK1/ERK1/2 signaling pathway, significantly accelerating bone formation. It exhibited excellent biocompatibility and bone repair potential.
	rGO-Ti	Kang et al. [117]	Graphene oxide (GO) is sonicated in water, then reduced with hydrazine hydrate to obtain reduced graphene oxide (rGO). rGO is uniformly coated on titanium (Ti) substrates using the MDD technique	The rGO coating enhances the hydrophilicity of the Ti substrate, promoting the proliferation and osteogenic differentiation of human mesenchymal stem cells (hMSCs). The material improves bone integration and has potential applications in orthopedics and dental implants.
	rGO-ST	Shin et al. [118]	Reduced graphene oxide (rGO) coating on SLA-treated titanium surfaces	rGO coating enhances osteogenic differentiation and osteointegration by improving surface wettability and protein adsorption. It promotes cell attachment, proliferation, and mineralization, leading to significantly increased bone formation and higher bone-to-implant contact (BIC) in vivo.

**Table 2:** Advantages and Disadvantages of Common Two-Dimensional Nanomaterials.

Material Category	Representative Materials	Main Advantages	Main Disadvantages
Graphene and Derivatives	Graphene (GO, rGO, GQDs, AGO, FG)	1. Extremely high specific surface area, enhancing drug loading capacity and surface reactivity; 2. High conductivity and thermal conductivity, suitable for photothermal therapy (PTT); 3. Good biocompatibility, can be functionalized for improved targeting; 4. Excellent antibacterial properties, particularly effective against *S. mutans* and other oral pathogens.	1. High production cost, especially for derivatives (e.g., GO), which tend to aggregate, affecting stability; 2. Long-term biodegradability issues, may cause immune response or toxicity; 3. Limited application in complex oral environments over long periods.
BP	Black Phosphorus Nanosheets (BPNSs), Nitrogen-Doped BP Quantum Dots (BPQDs)	1. Good biodegradability, degradation products are non-toxic phosphates that aid bone regeneration; 2. Strong photothermal conversion ability, suitable for photothermal therapy (PTT); 3. Photodynamic therapy (PDT) efficacy; 4. Strong antioxidant capacity, regulates immune response.	1. Poor photothermal stability, easy to degrade in air; 2. Excessive degradation may lead to short-lived therapeutic effects; 3. Synthesis process may introduce impurities, affecting biological safety.
MXene	Ti_3_C_2_T_x_, Nb_2_C, etc.	1. High conductivity, good hydrophilicity, suitable for drug delivery; 2. Strong photothermal properties, suitable for photothermal therapy (PTT); 3. Effectively scavenges ROS, has antioxidant and immunomodulatory functions; 4. High surface reactivity, suitable for surface modification and functionalization.	1. Insufficient biodegradability and long-term stability, may release metal ions leading to toxicity; 2. May cause immune response in certain environments; 3. Difficult to control high-efficiency preparation and stability.
LDHs	ZnAl-LDH, CuAl-LDH, MgFe-LDH, etc.	1. Releases various metal ions, regulates immune response; 2. Good biocompatibility, suitable for long-term drug delivery; 3. High surface area and ion exchange capacity, enhancing drug loading and release.	1. Ion release may cause accumulation in the body; 2. Poor stability in aqueous environments, may lead to degradation issues; 3. Long-term safety and stability need further validation.
TMDs	MoS_2_, WS_2_, etc.	1. Efficient photothermal conversion, suitable for photothermal therapy (PTT); 2. Can be surface-modified to enhance drug delivery functions; 3. Synergizes with other therapies (e.g., PDT) to enhance efficacy; 4. Has antioxidant properties, scavenges ROS.	1. May have structural defects during preparation, affecting performance; 2. Photothermal efficiency is influenced by material size and surface conditions; 3. Photostability and biocompatibility need further research.
2D MOFs	Fe_2_O_3_-Porphyrin MOF, etc.	1. Porous structure with high drug loading capacity; 2. Drug release can be controlled through functionalization and metal node modulation; 3. Strong photothermal and photodynamic therapy capabilities, suitable for targeted treatment; 4. Tunable surface chemistry, easy for surface modification.	1. Complex preparation processes, difficult for large-scale production; 2. Poor water stability, may degrade in the oral environment; 3. Biocompatibility is not fully validated, posing challenges for clinical translation.
